# Supplementary material for: CCDC32 stabilizes clathrin-coated pits and drives their invagination
Source: eLife. 2026 Jan 5;14:RP107039. doi: 10.7554/eLife.107039 (PMC12768407; doi:10.7554/eLife.107039)
Supplement: Figure 1—figure supplement 1—source data 1. [file elife-107039-fig1-figsupp1-data1.zip › Figure 1-figure supplement 1-source data 1/Figure supplement 1-Source Data 1.pdf]

Figure supplement 1A

Vinculin

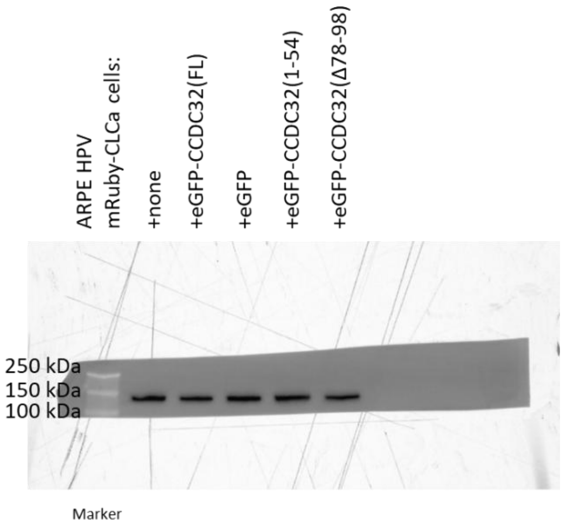

GFP

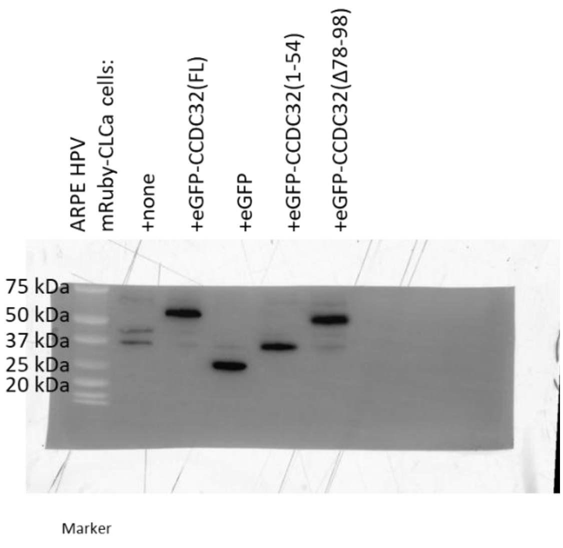

Figure supplement 1B

Vinculin

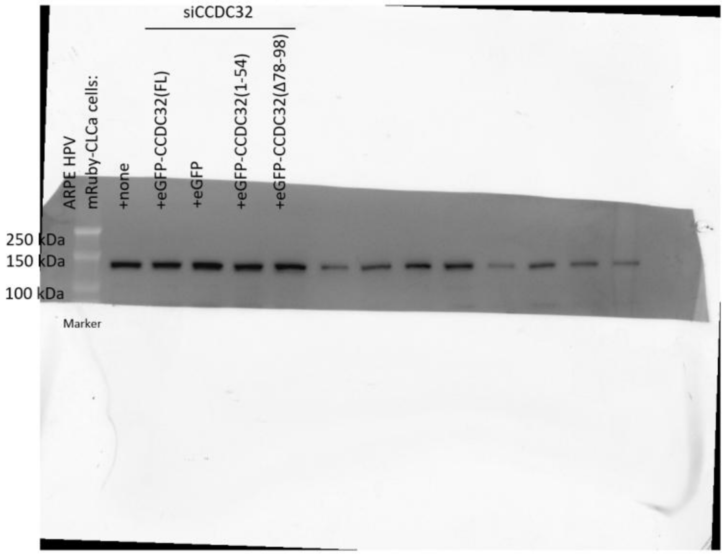

CCDC32

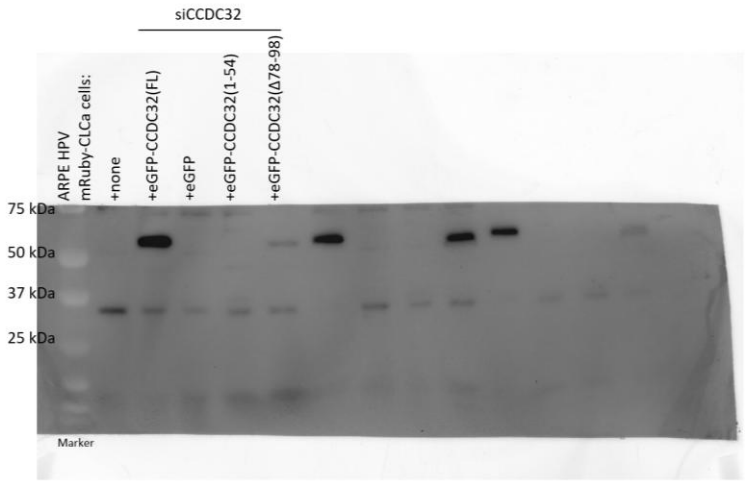

Figure supplement 1

(A) Western blotting indicates similar expression levels of exogenous eGFP-CCDC32(FL), eGFP, eGFP-CCDC32(1-54) and eGFP-CCDC32(Δ78-98) in ARPE-HPV mRuby-CLCa cells. (B) siCCDC32 knocked down endogenous CCDC32 but not exogenously introduced eGFP-CCDC32(FL) from ARPE-HPV mRuby-CLCa cells. Note that the anti-CCDC32 antibody does not detect the eGFP-CCDC32(Δ78-98) as well as full-length and is unable to detect eGFP-CCDC32(1-54).
