# Supplementary figures and images for: CCDC32 stabilizes clathrin-coated pits and drives their invagination
Source: eLife. 2026 Jan 5;14:RP107039. doi: 10.7554/eLife.107039 (PMC12768407; doi:10.7554/eLife.107039)

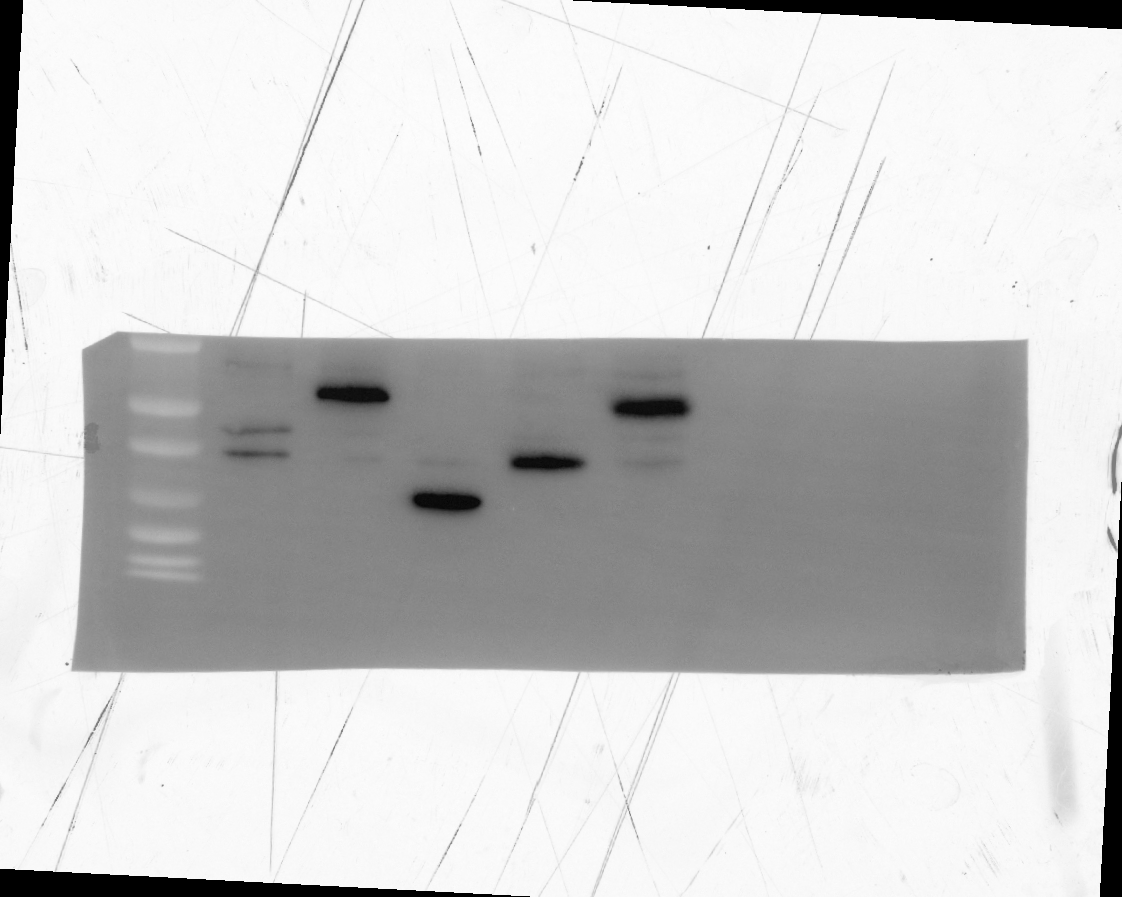

Supplement: Figure 1—figure supplement 1—source data 2. [file elife-107039-fig1-figsupp1-data2.zip › Figure 1-figure supplement 1-source data 3/Supplement 1A-GFP.tif]

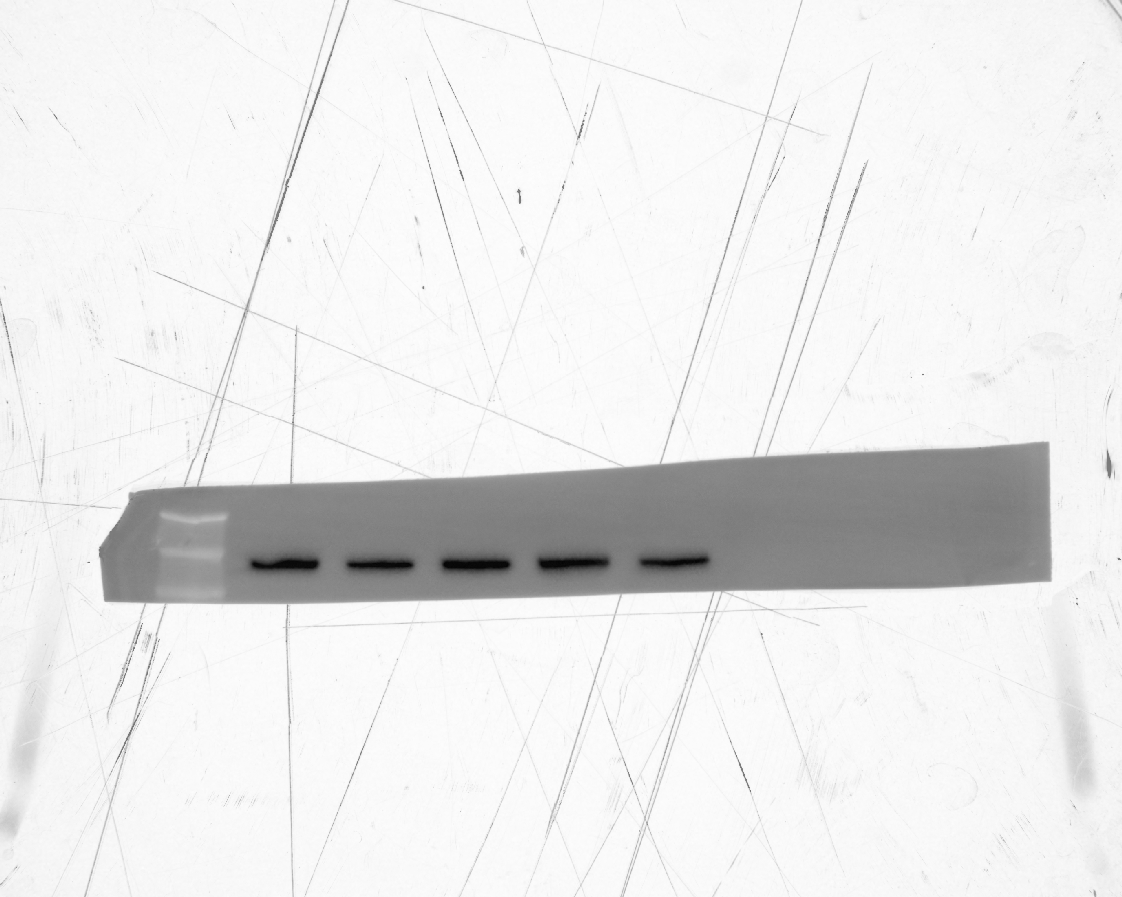

Supplement: Figure 1—figure supplement 1—source data 2. [file elife-107039-fig1-figsupp1-data2.zip › Figure 1-figure supplement 1-source data 3/Supplement 1A-vinculin.tif]

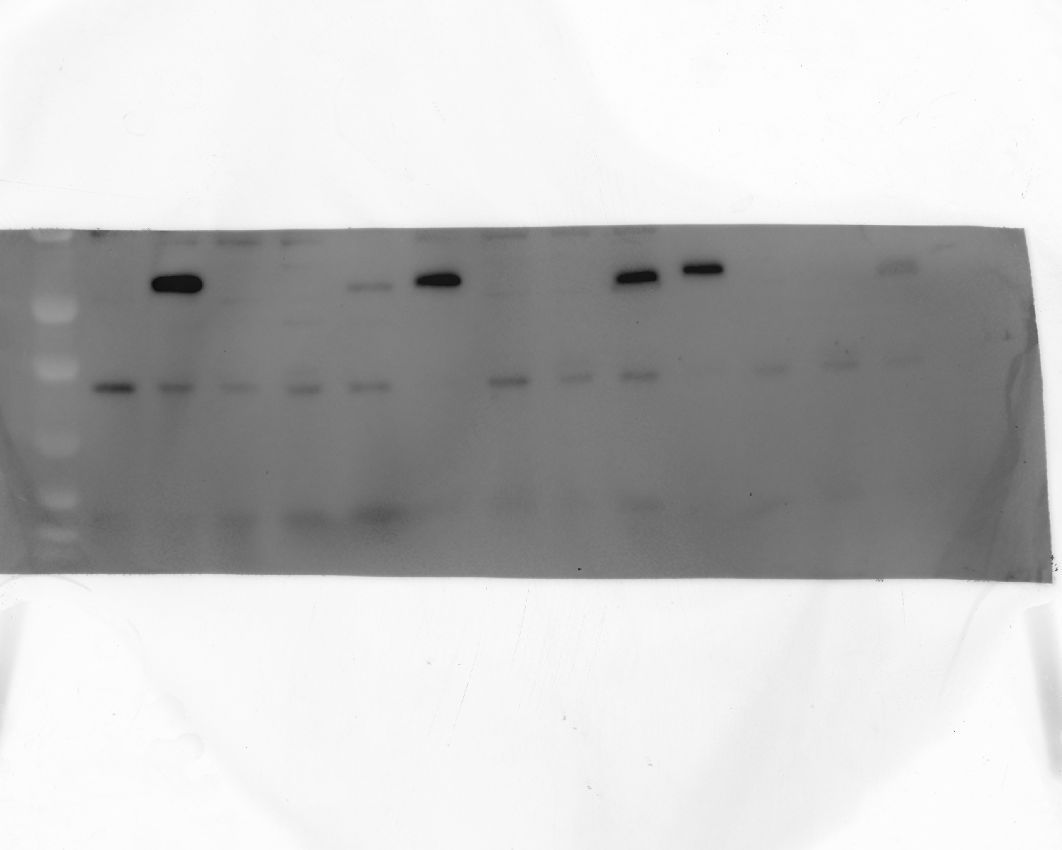

Supplement: Figure 1—figure supplement 1—source data 2. [file elife-107039-fig1-figsupp1-data2.zip › Figure 1-figure supplement 1-source data 3/Supplement 1B-GFP.tif]

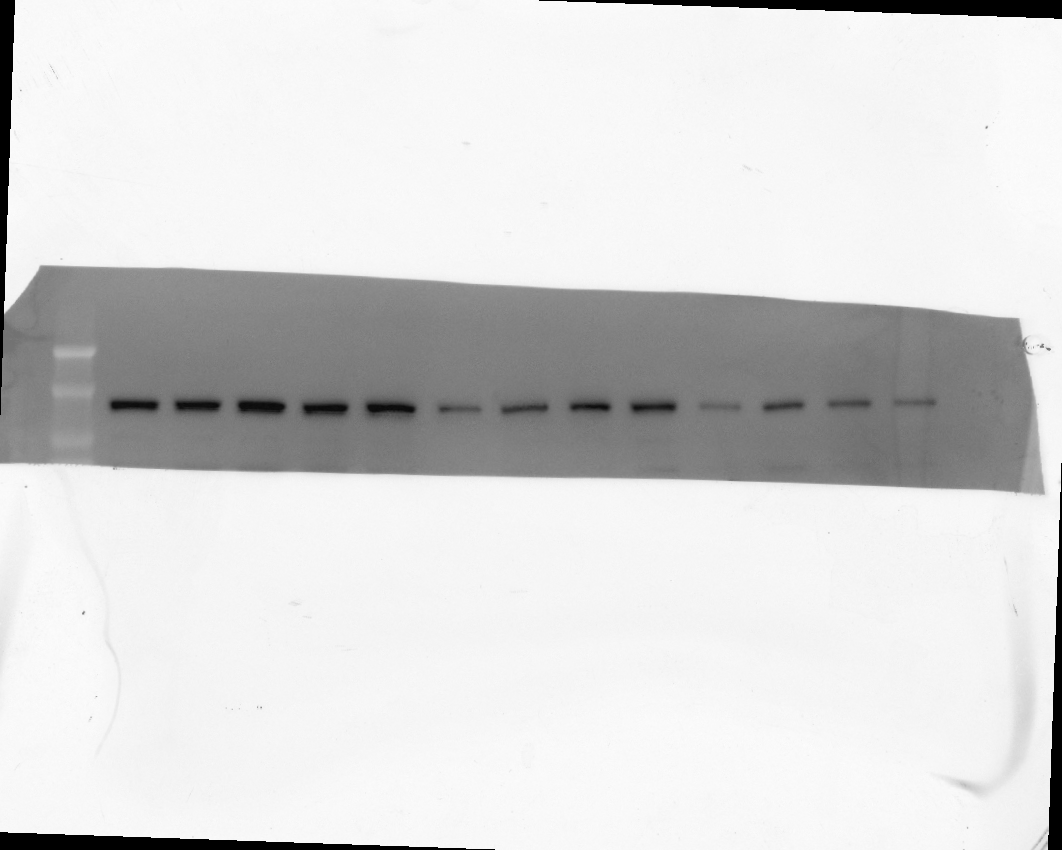

Supplement: Figure 1—figure supplement 1—source data 2. [file elife-107039-fig1-figsupp1-data2.zip › Figure 1-figure supplement 1-source data 3/Supplement 1B-vinculin.tif]

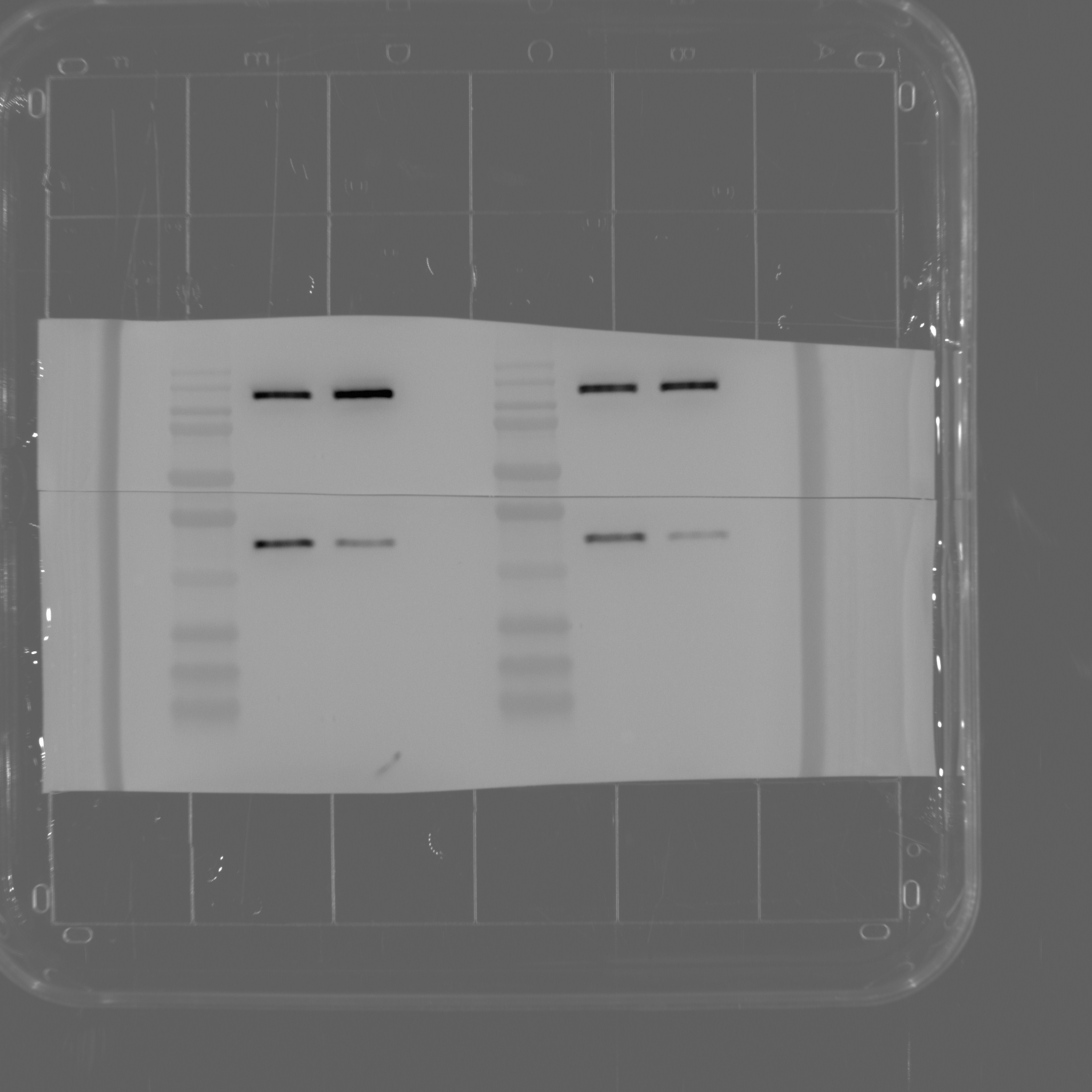

Supplement: Figure 2—source data 3. [file elife-107039-fig2-data3.zip › Figure 2-source data 3/2A.tif]

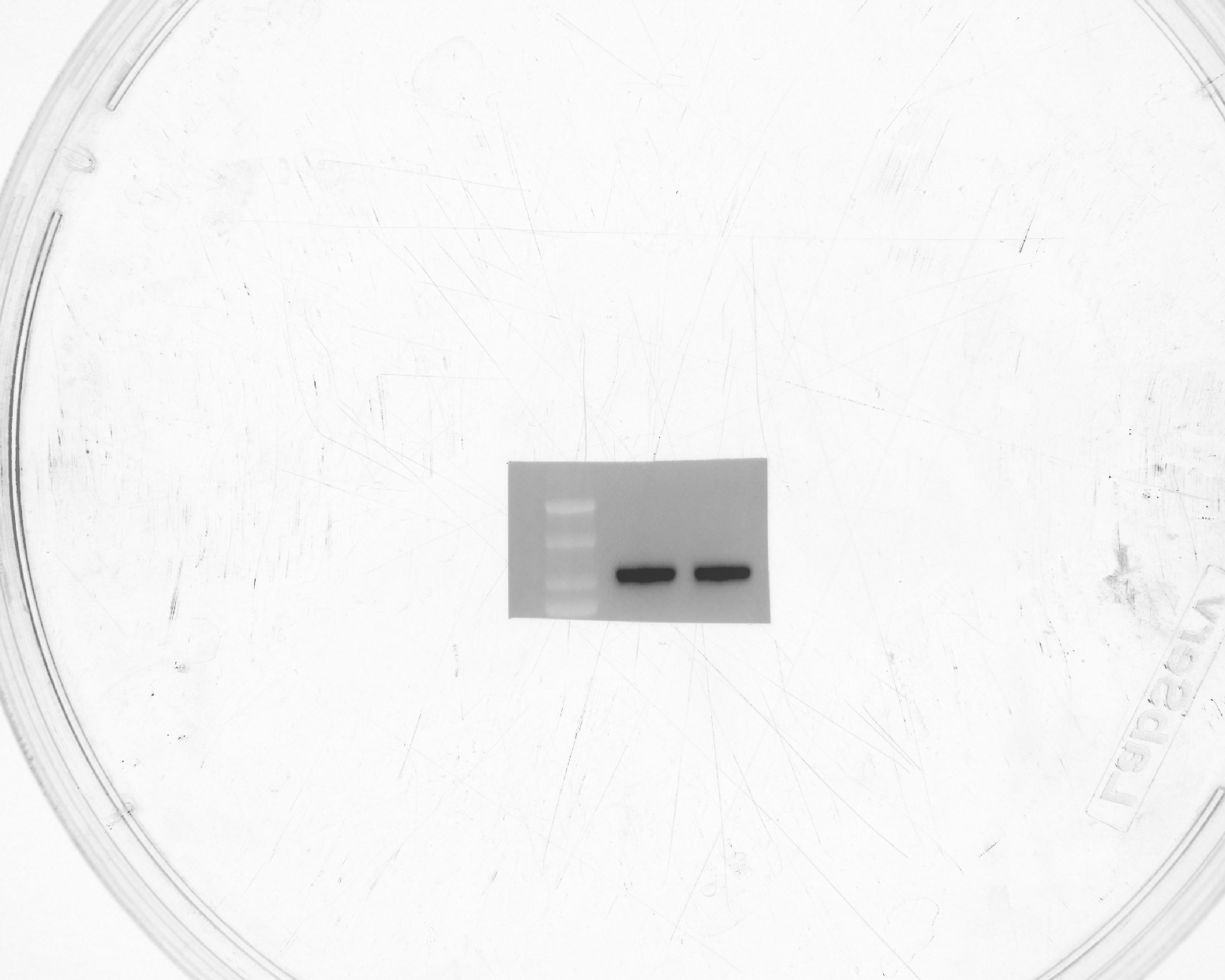

Supplement: Figure 2—figure supplement 1—source data 3. [file elife-107039-fig2-figsupp1-data3.zip › Figure 2-figure supplement 1-source data 3/supplement 1A-AP2-alpha.tif]

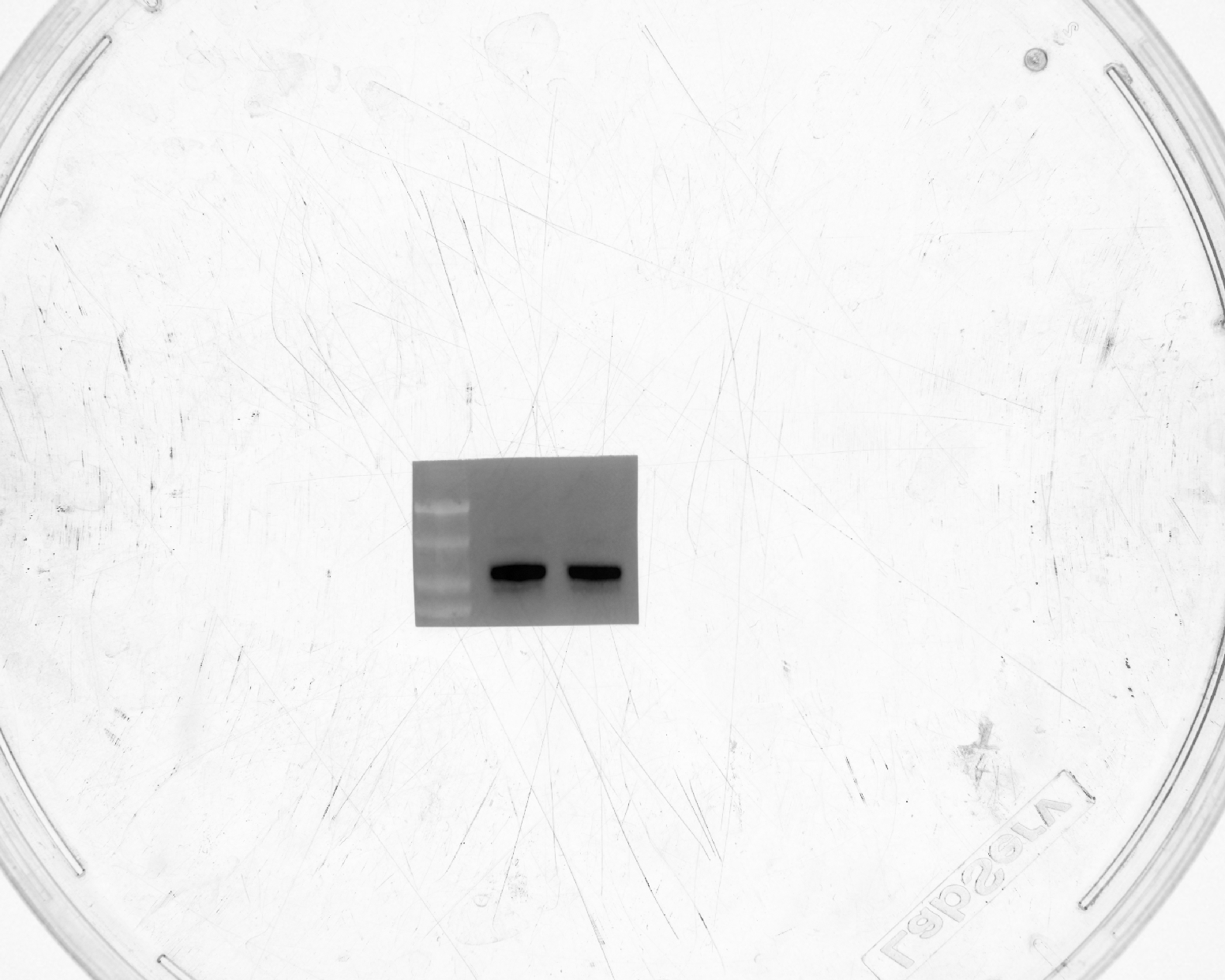

Supplement: Figure 2—figure supplement 1—source data 3. [file elife-107039-fig2-figsupp1-data3.zip › Figure 2-figure supplement 1-source data 3/supplement 1A-AP2-beta.tif]

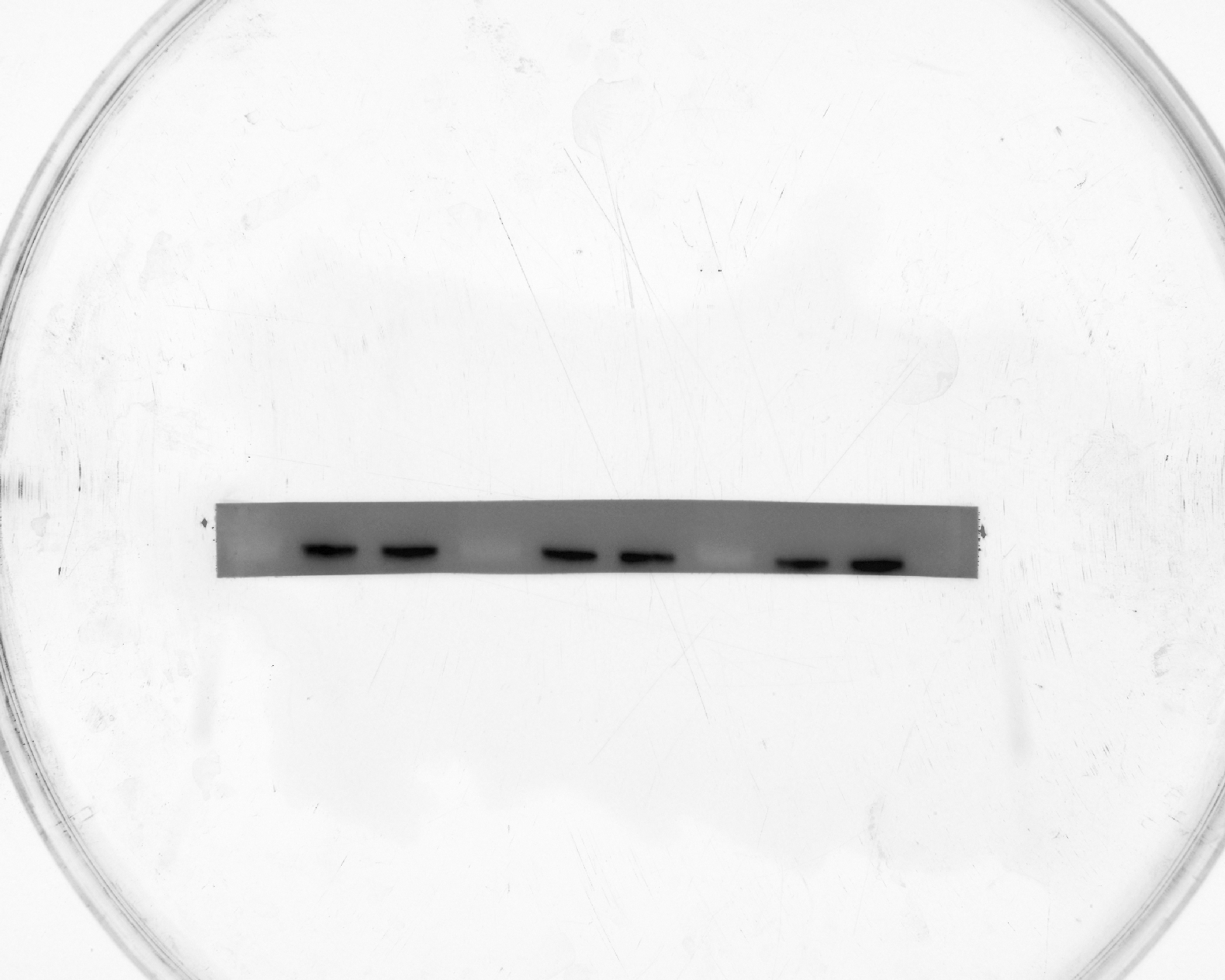

Supplement: Figure 2—figure supplement 1—source data 3. [file elife-107039-fig2-figsupp1-data3.zip › Figure 2-figure supplement 1-source data 3/supplement 1A-AP2-mu.tif]

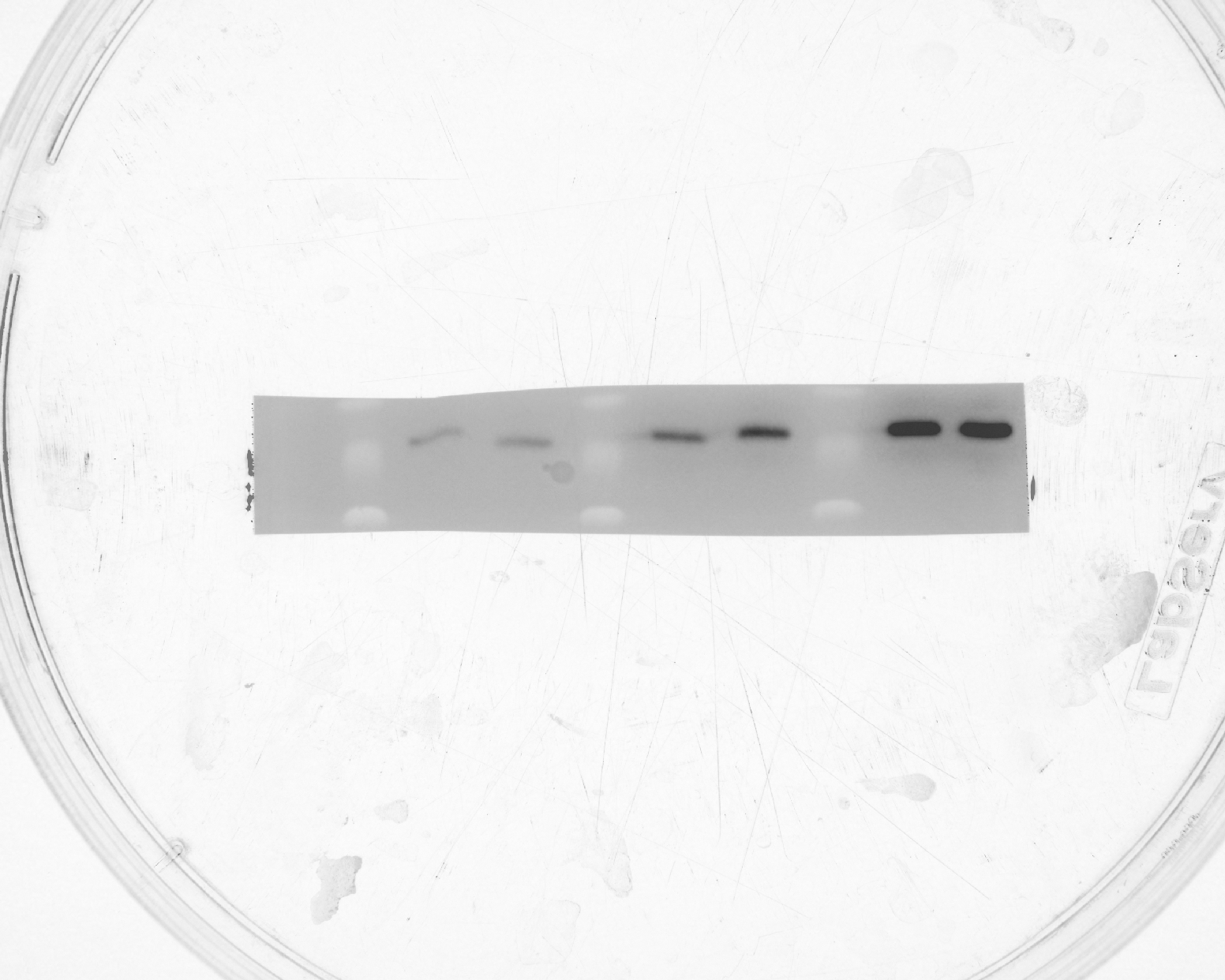

Supplement: Figure 2—figure supplement 1—source data 3. [file elife-107039-fig2-figsupp1-data3.zip › Figure 2-figure supplement 1-source data 3/supplement 1A-AP2-sigma.tif]

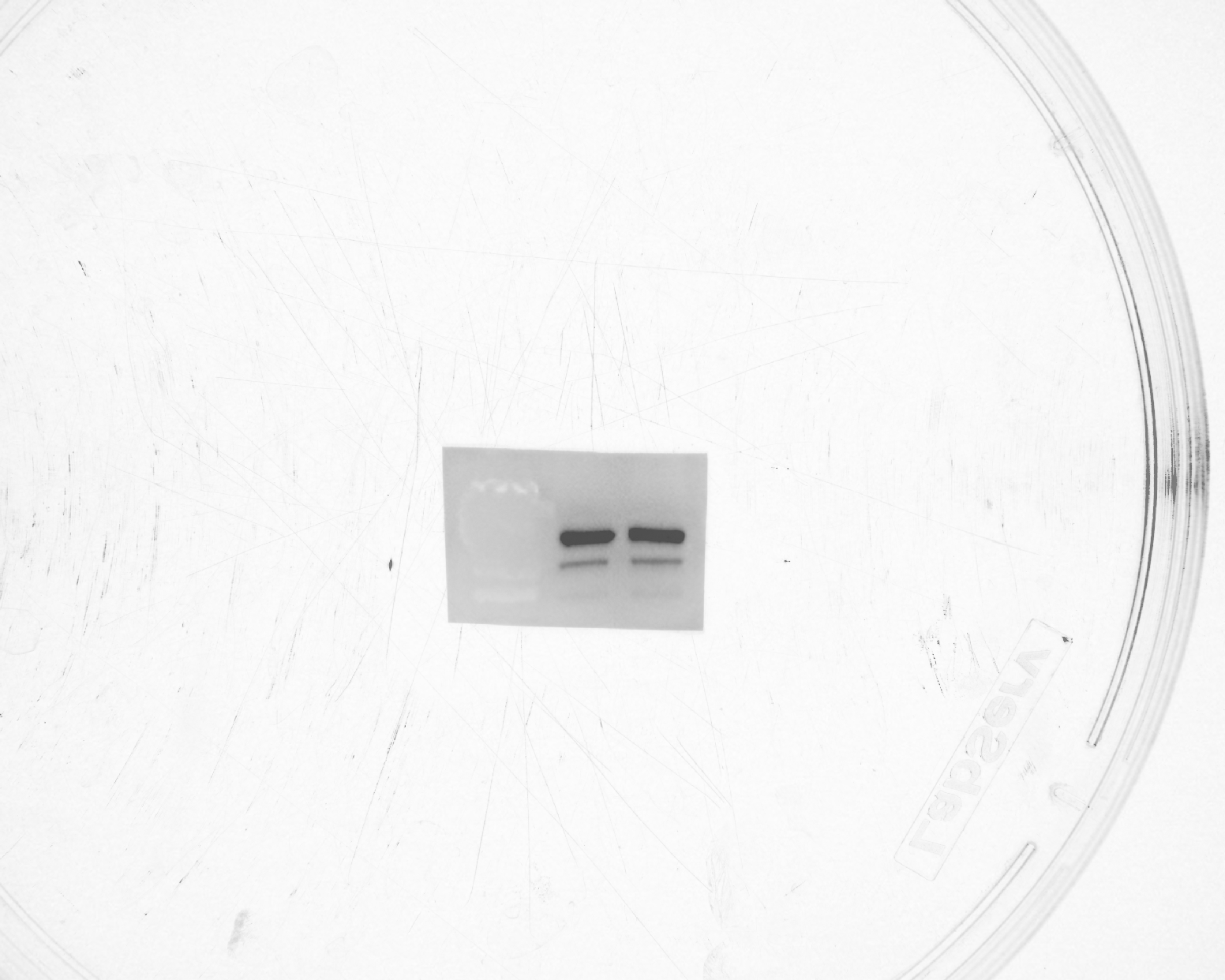

Supplement: Figure 2—figure supplement 1—source data 3. [file elife-107039-fig2-figsupp1-data3.zip › Figure 2-figure supplement 1-source data 3/supplement 1A-Vinculin.tif]

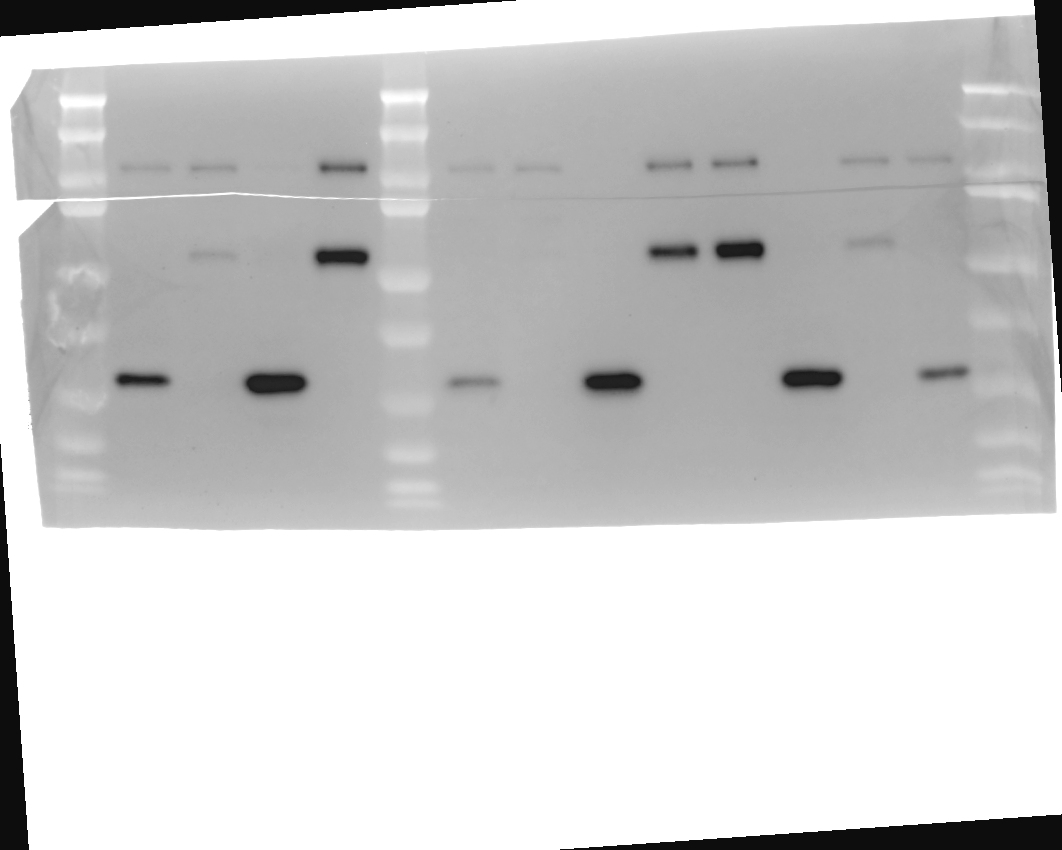

Supplement: Figure 5—source data 4. [file elife-107039-fig5-data4.zip › Figure 5-source data 4/5E.tif]

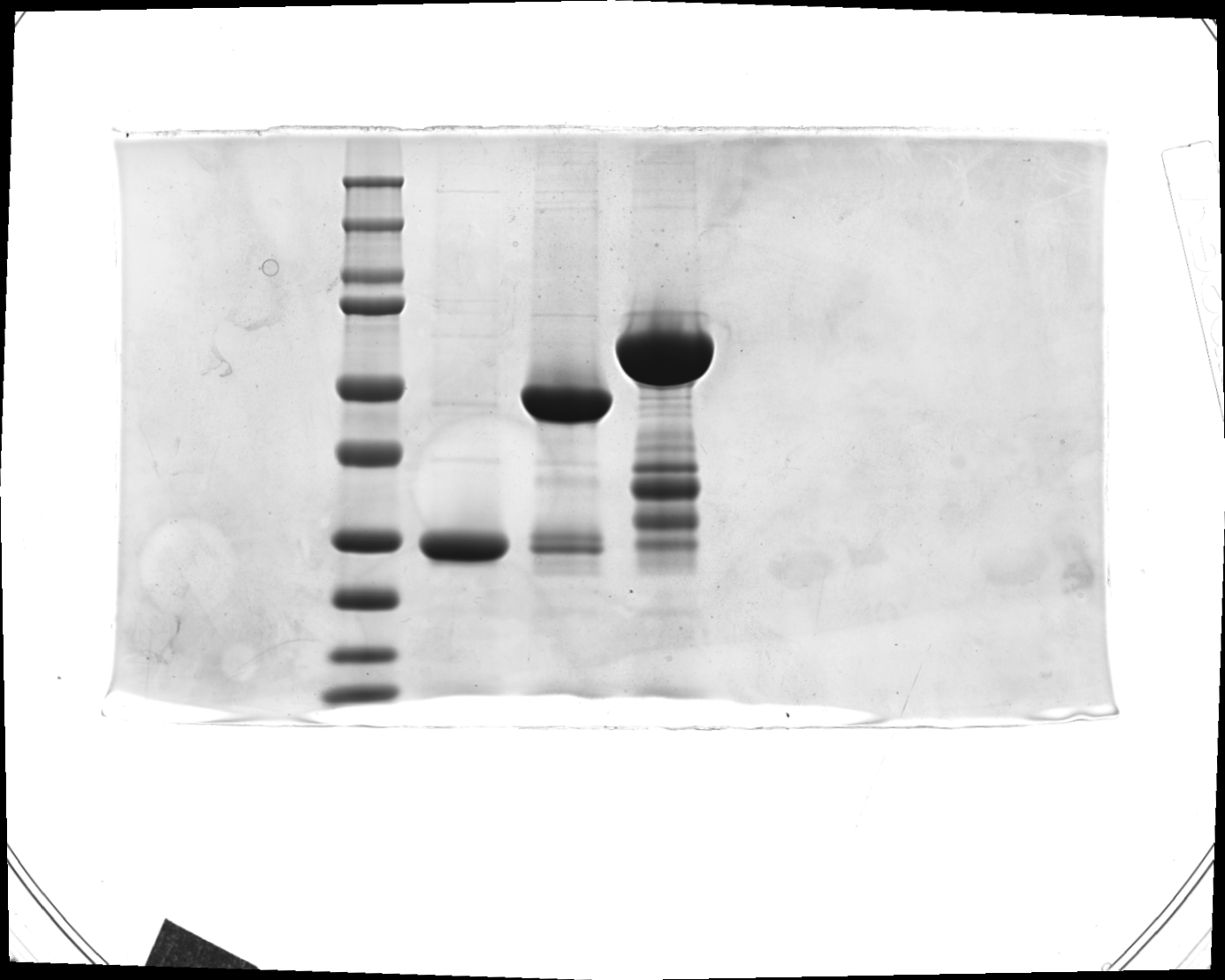

Supplement: Figure 5—source data 4. [file elife-107039-fig5-data4.zip › Figure 5-source data 4/5G.tif]

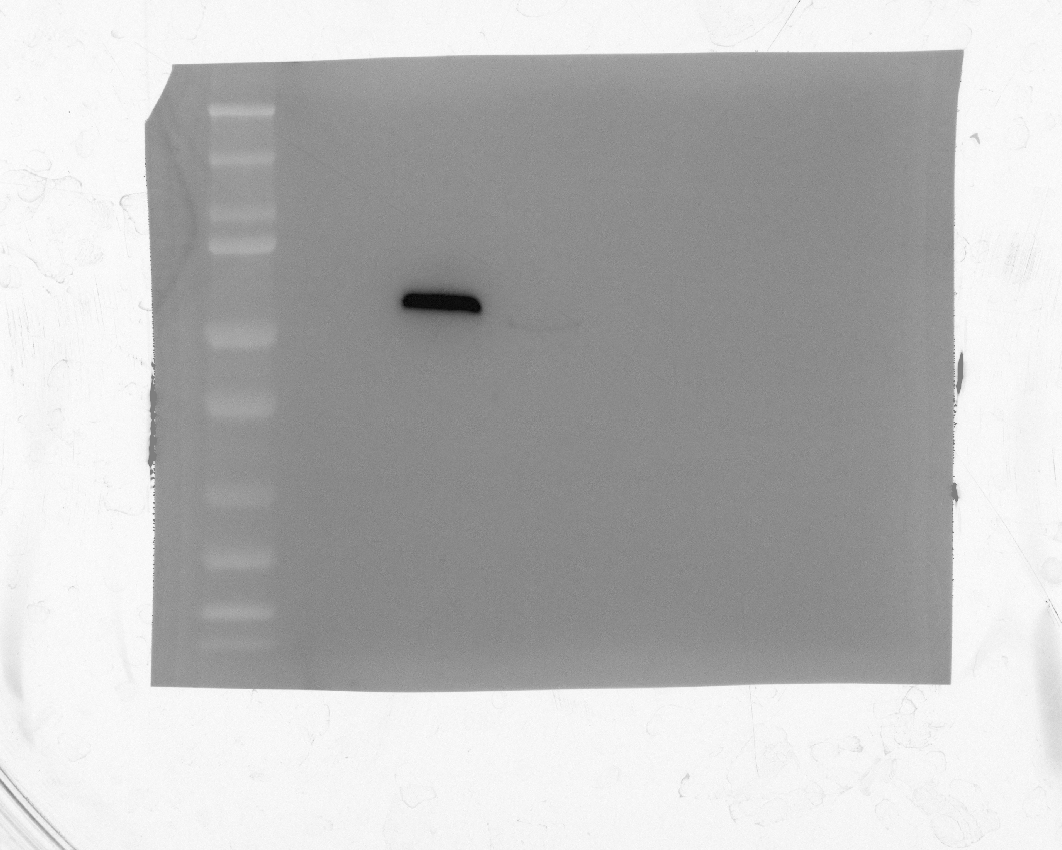

Supplement: Figure 5—source data 4. [file elife-107039-fig5-data4.zip › Figure 5-source data 4/5H.tif]

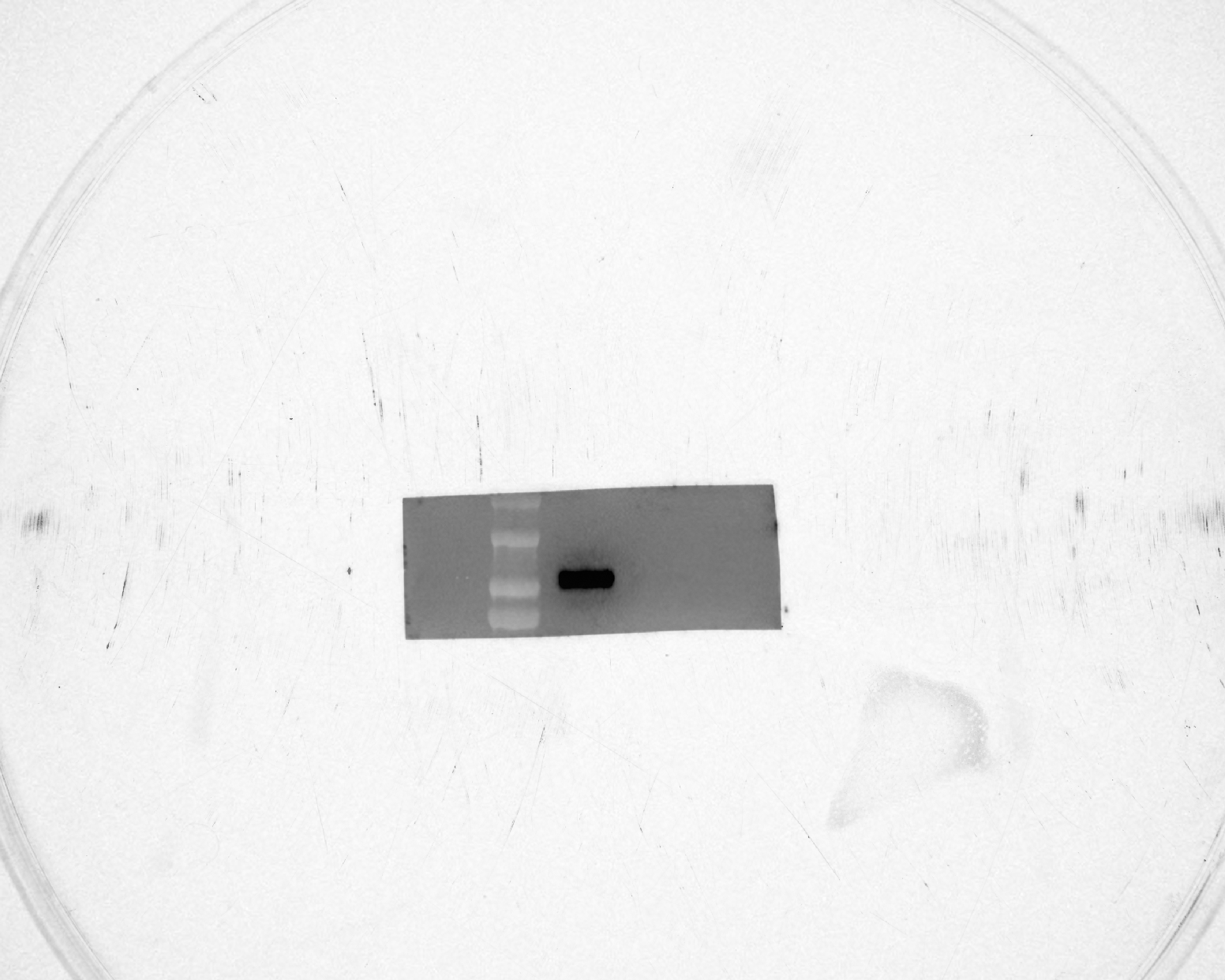

Supplement: Figure 5—figure supplement 1—source data 2. [file elife-107039-fig5-figsupp1-data2.zip › Figure 5-figure supplement 1-source data 2/supplement 1- AP2-alpha.tif]

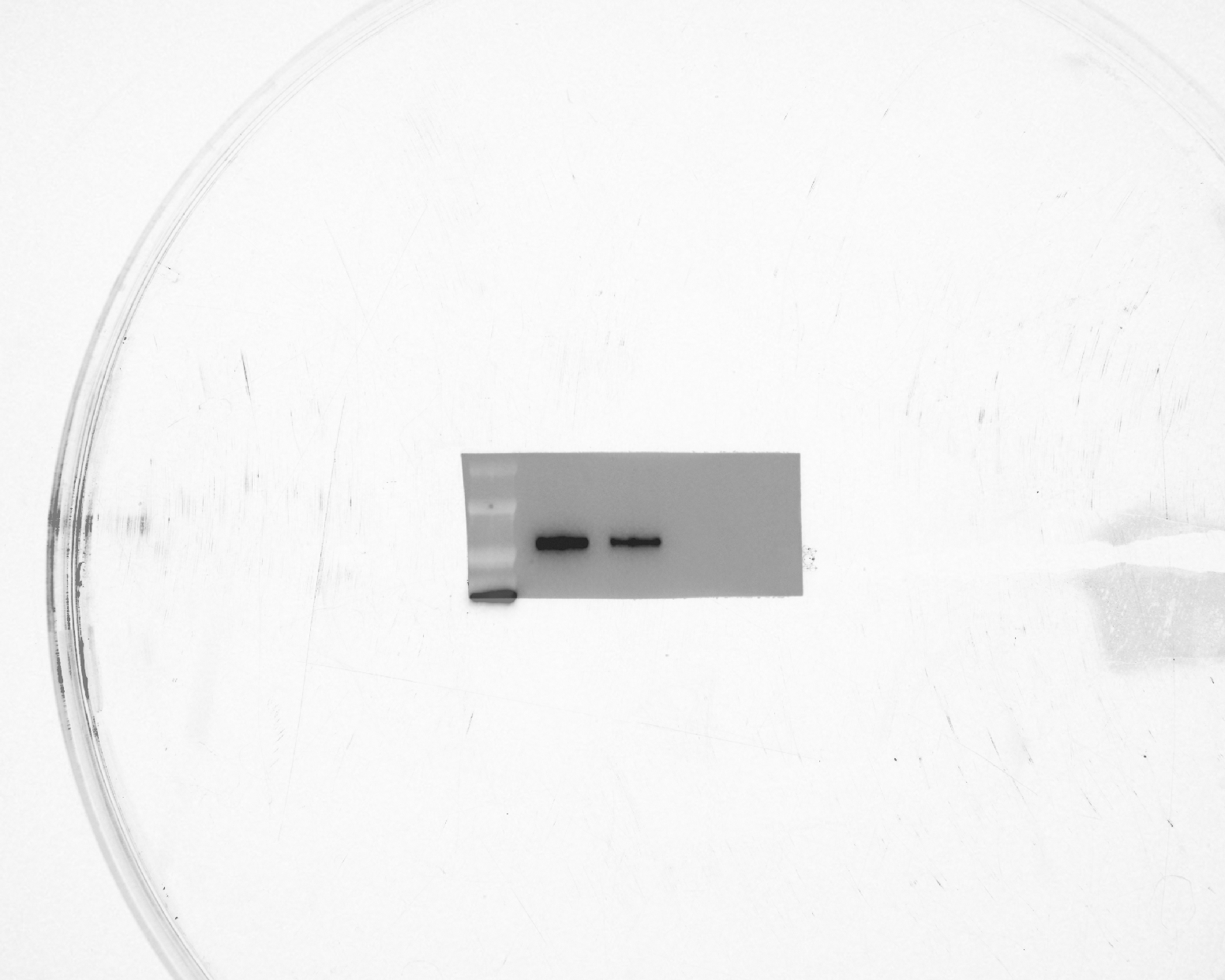

Supplement: Figure 5—figure supplement 1—source data 2. [file elife-107039-fig5-figsupp1-data2.zip › Figure 5-figure supplement 1-source data 2/supplement 1- AP2-beta.tif]

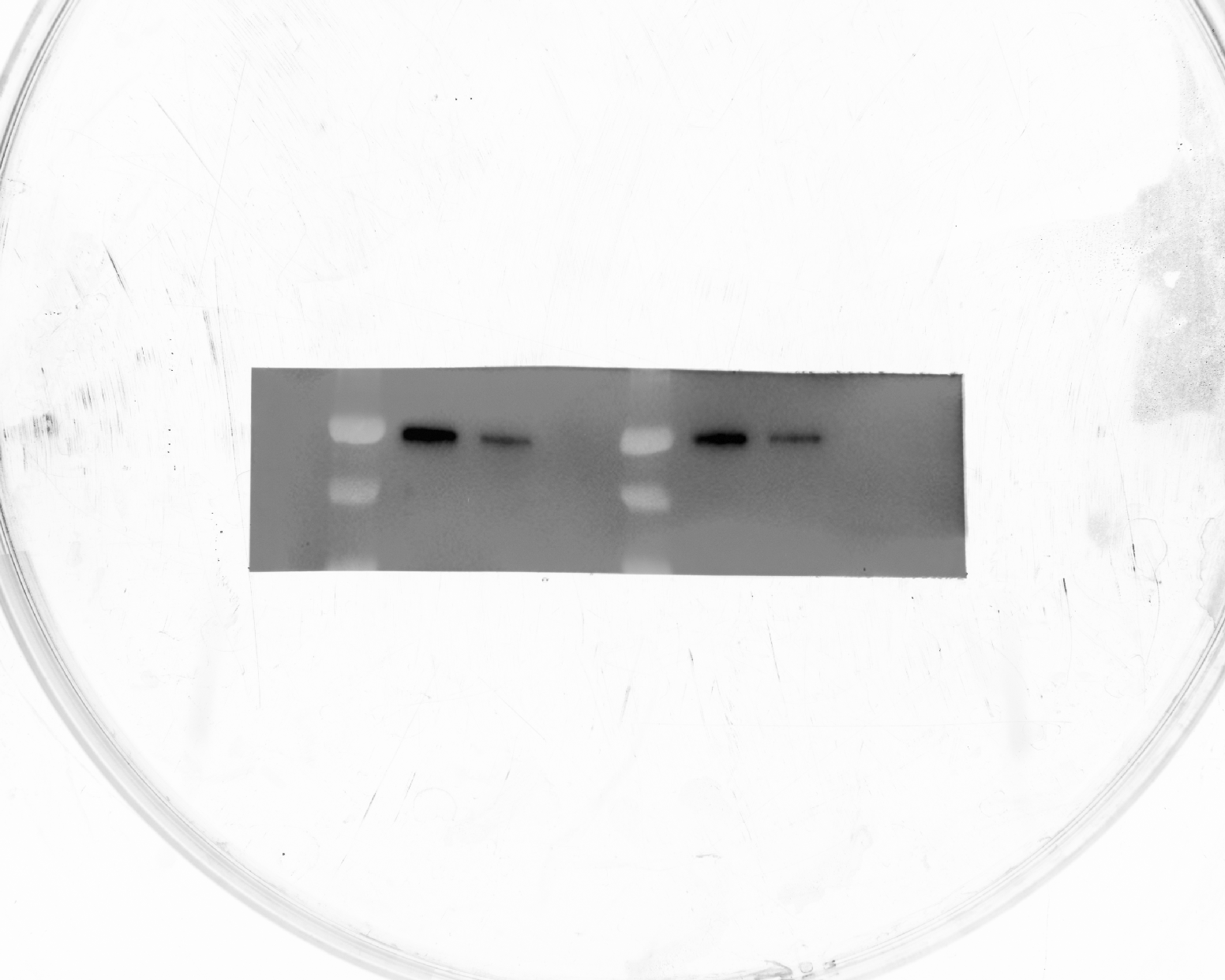

Supplement: Figure 5—figure supplement 1—source data 2. [file elife-107039-fig5-figsupp1-data2.zip › Figure 5-figure supplement 1-source data 2/supplement 1-AP2-mu.tif]

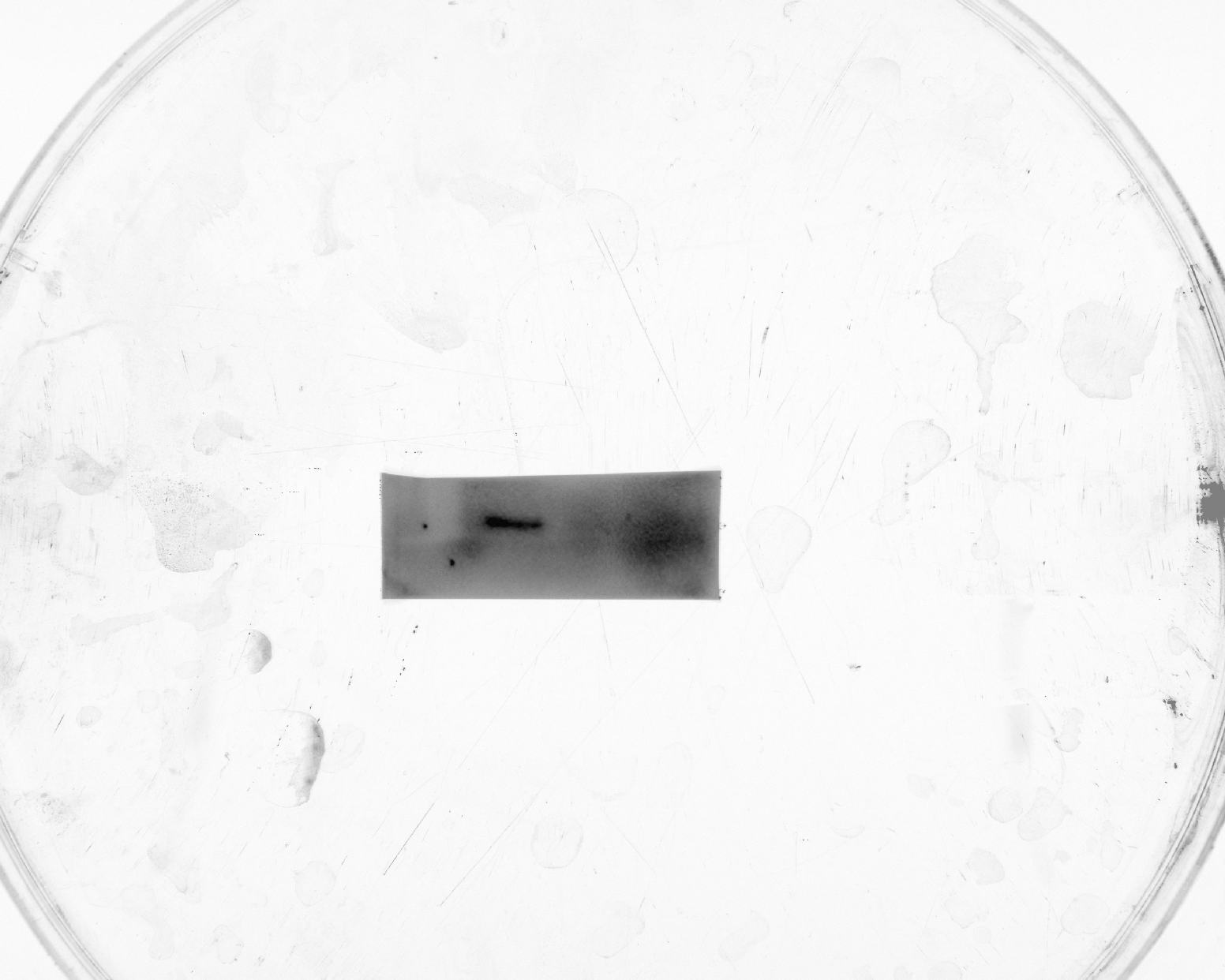

Supplement: Figure 5—figure supplement 1—source data 2. [file elife-107039-fig5-figsupp1-data2.zip › Figure 5-figure supplement 1-source data 2/supplement 1-AP2-sigma.tif]

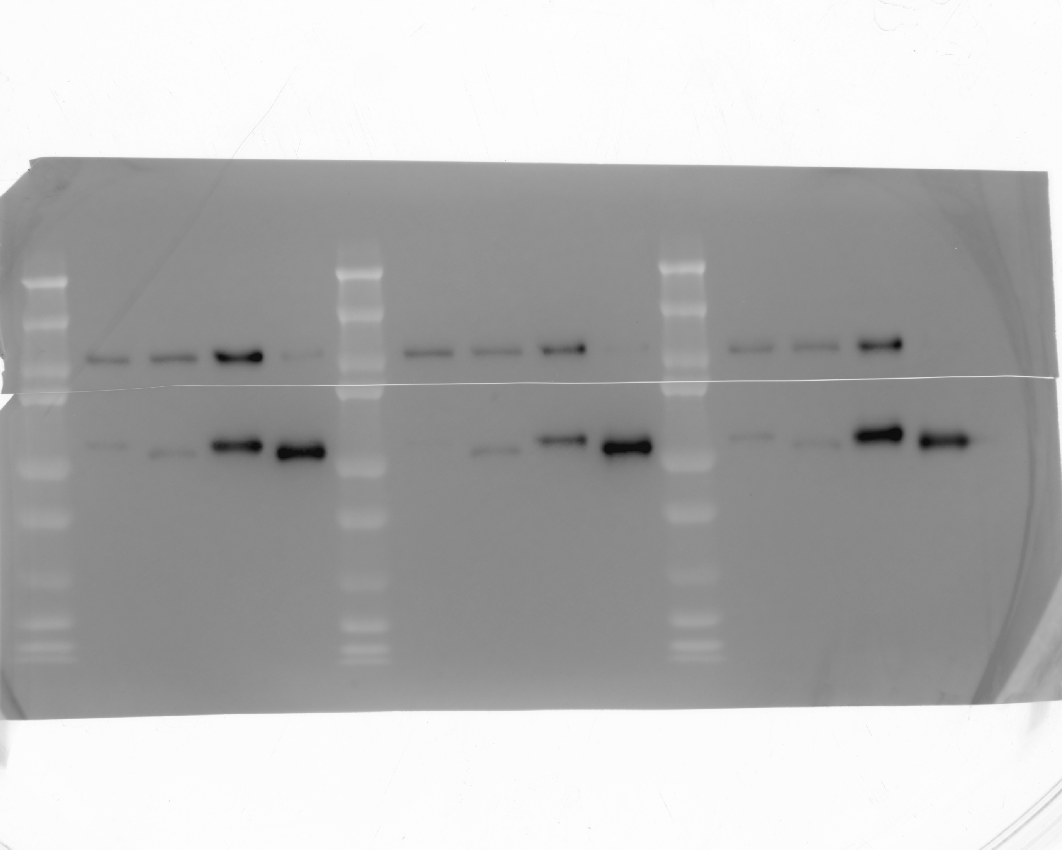

Supplement: Figure 6—source data 3. [file elife-107039-fig6-data3.zip › Figure 6-source data 3/6B-left.tif]

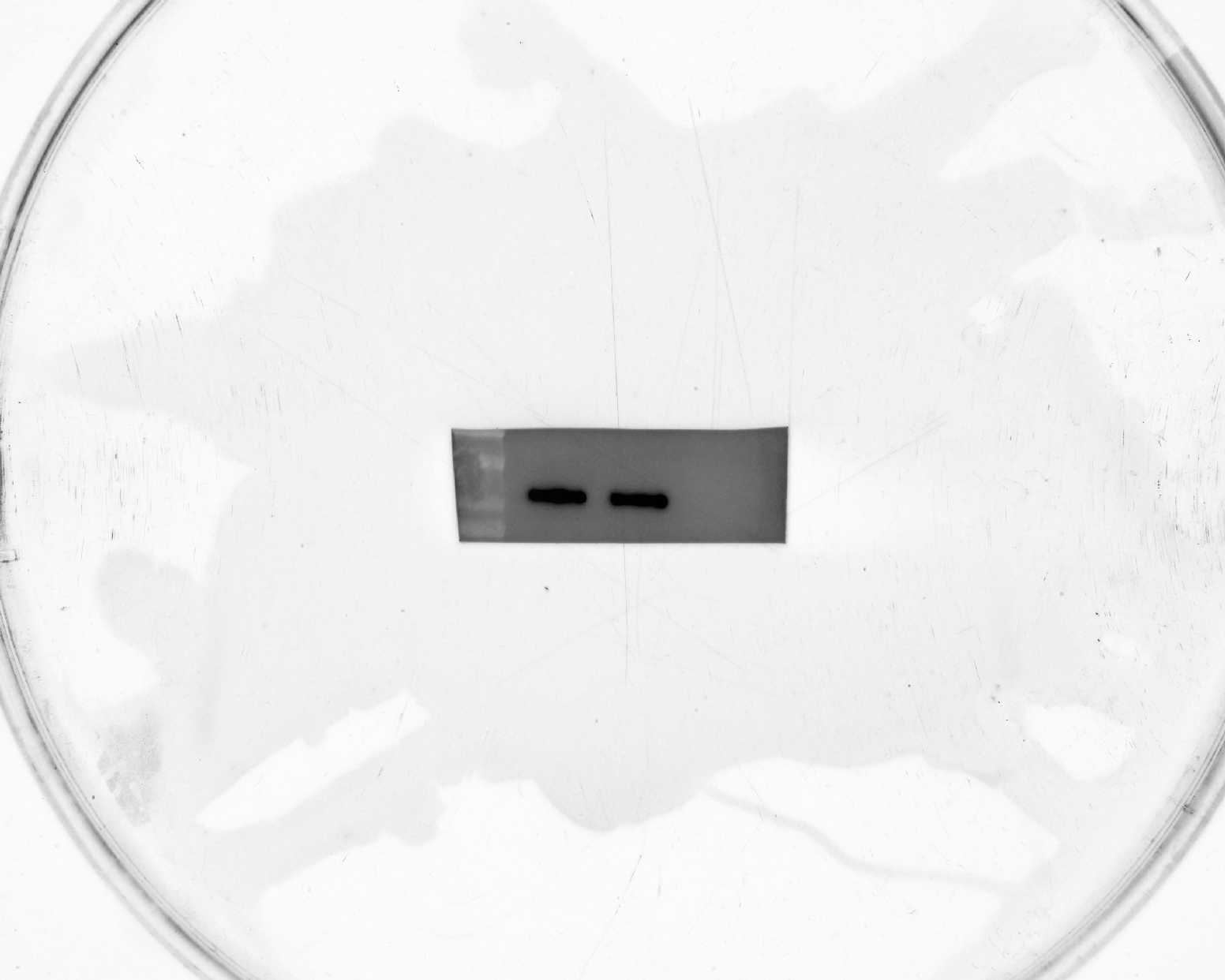

Supplement: Figure 6—source data 3. [file elife-107039-fig6-data3.zip › Figure 6-source data 3/6B-right-beta.tif]

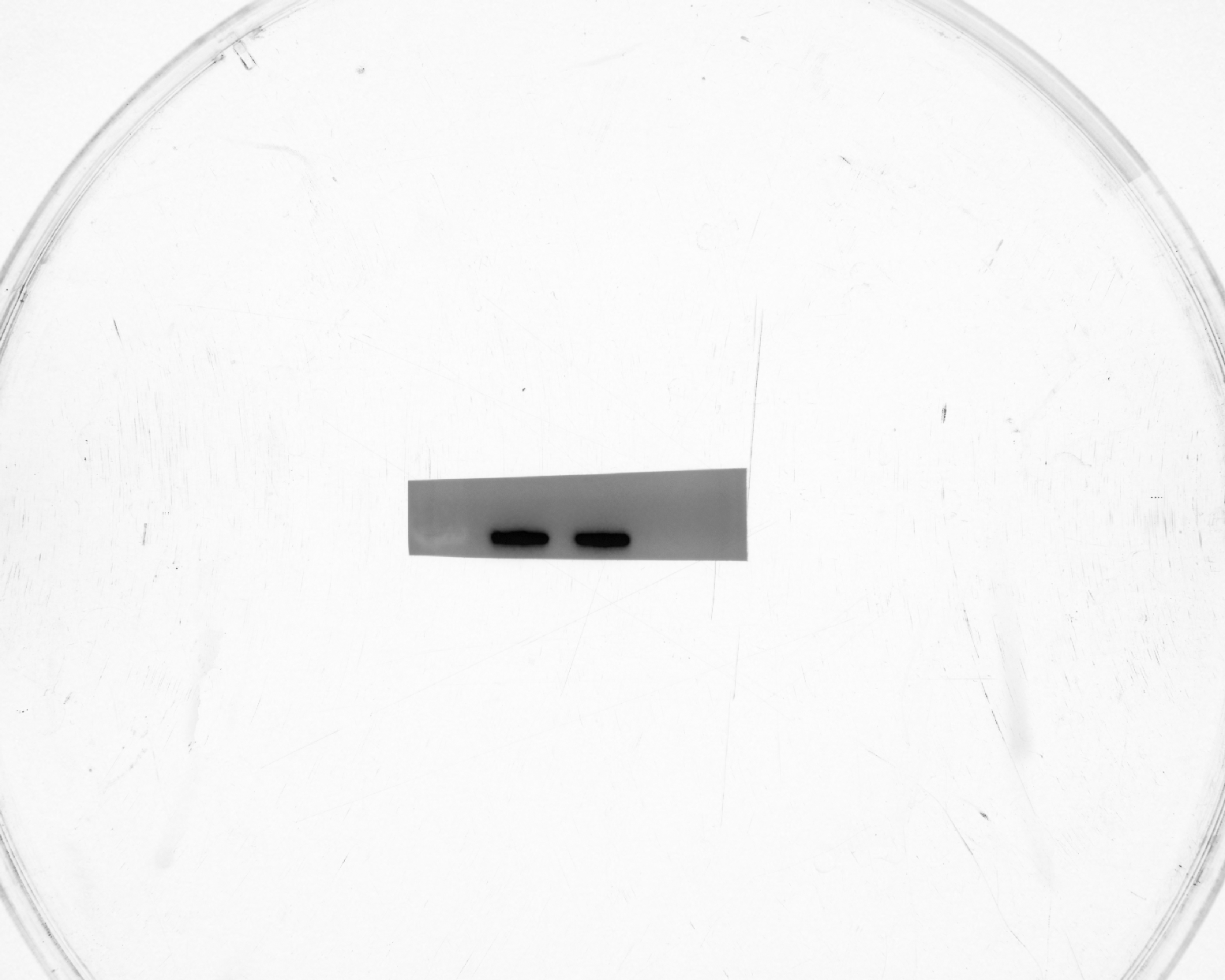

Supplement: Figure 6—source data 3. [file elife-107039-fig6-data3.zip › Figure 6-source data 3/6B-right-mu.tif]

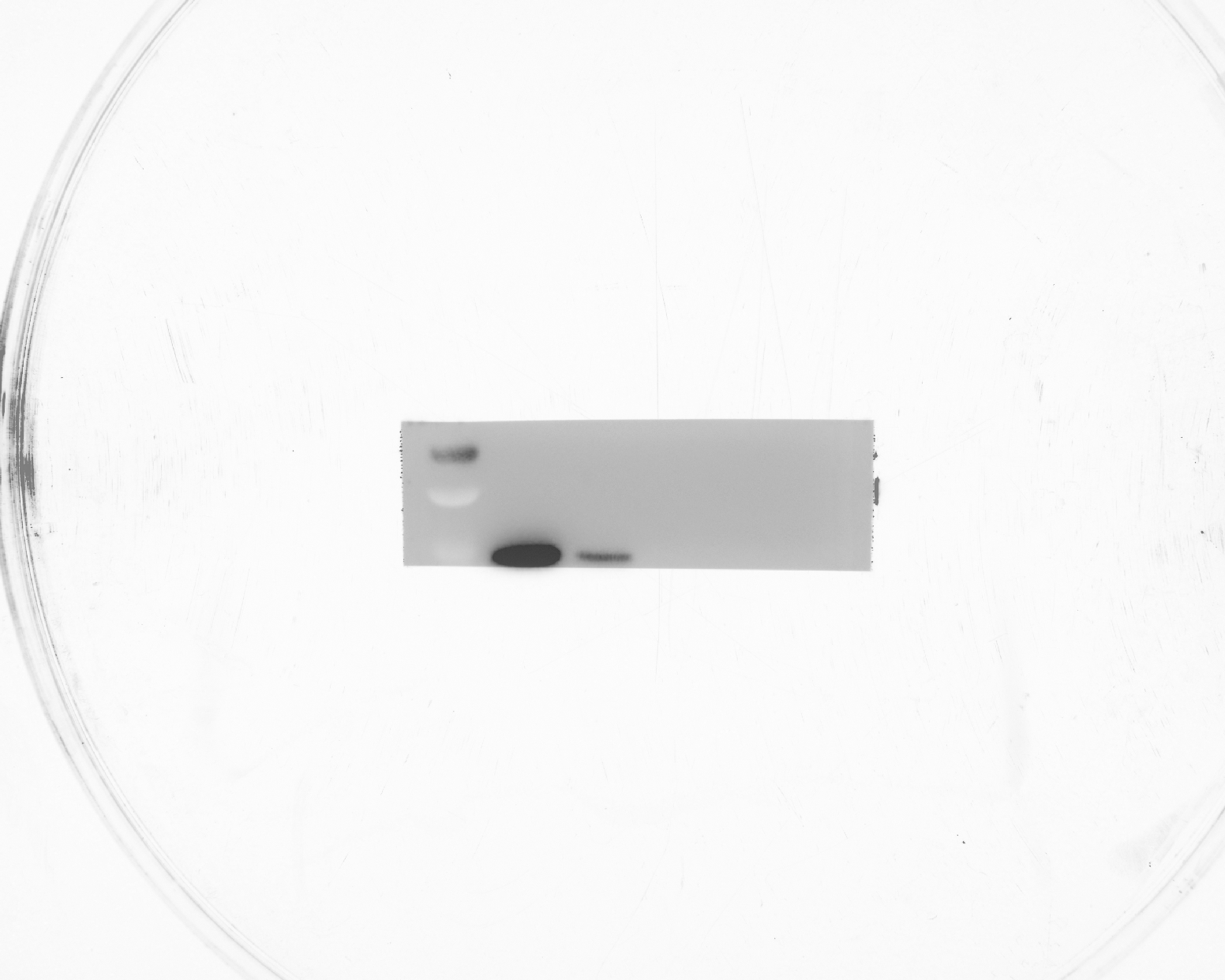

Supplement: Figure 6—source data 3. [file elife-107039-fig6-data3.zip › Figure 6-source data 3/6B-right-sigma.tif]

## supplement 1C

### CCDC32

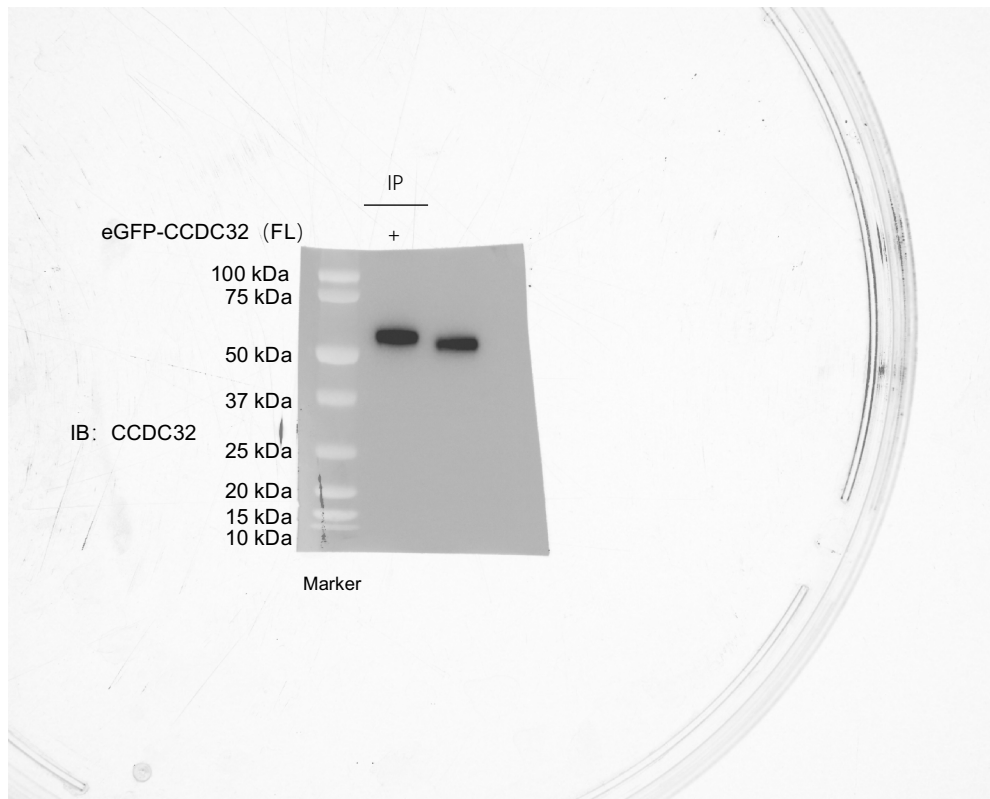

## supplement 1

(C) co-IP of eGFP-CCDC32 did not pulldown endogenous CCDC32.

Supplement: Figure 6—figure supplement 1—source data 1. [file elife-107039-fig6-figsupp1-data1.zip › Figure 6-figure supplement 1-source data 1/Figure 6-figure supplement 6-source data 1.pdf]

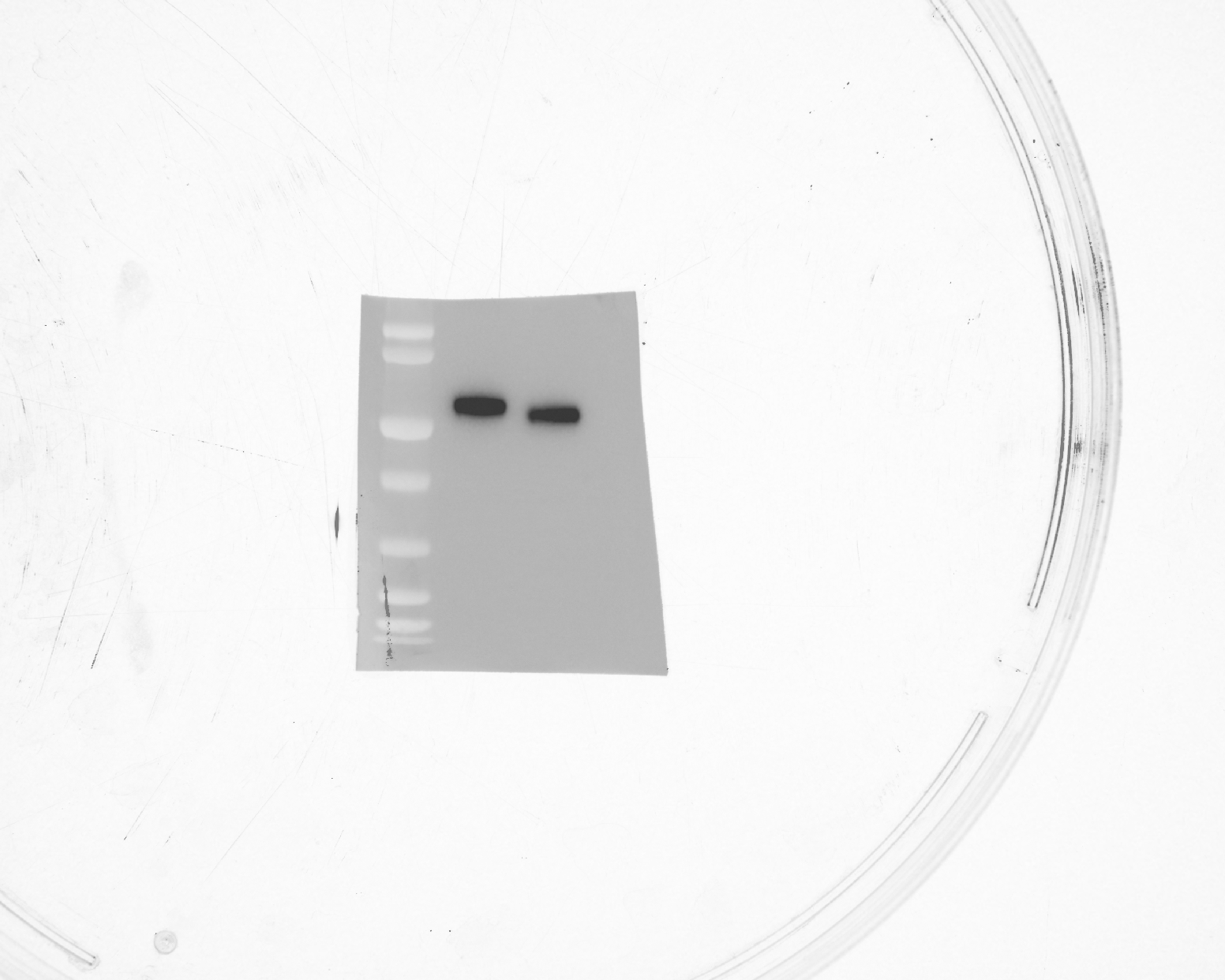

Supplement: Figure 6—figure supplement 1—source data 2. [file elife-107039-fig6-figsupp1-data2.zip › Figure 6-figure supplement 1-source data 2/supplemet 1C.tif]

7B

AP2- $\alpha$ 、GFP

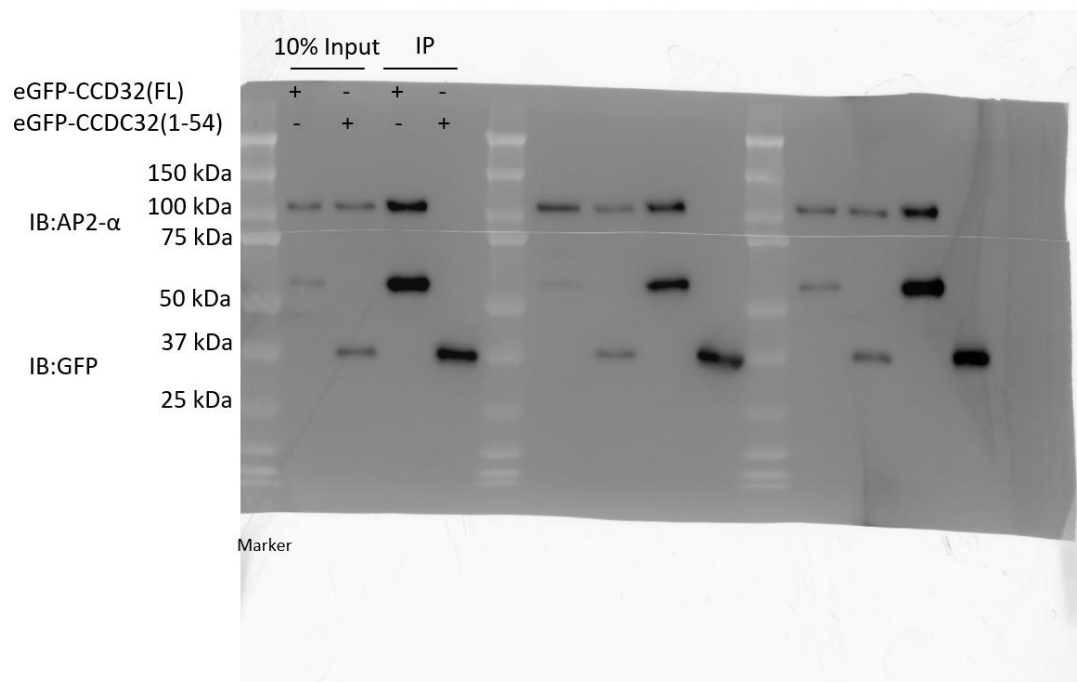

Figure 7

(B) Representative immunoblotting result of n=3 IP samples.

Supplement: Figure 7—source data 2. [file elife-107039-fig7-data2.zip › Figure 7-source data 2/Figure 7-source data 2.pdf]

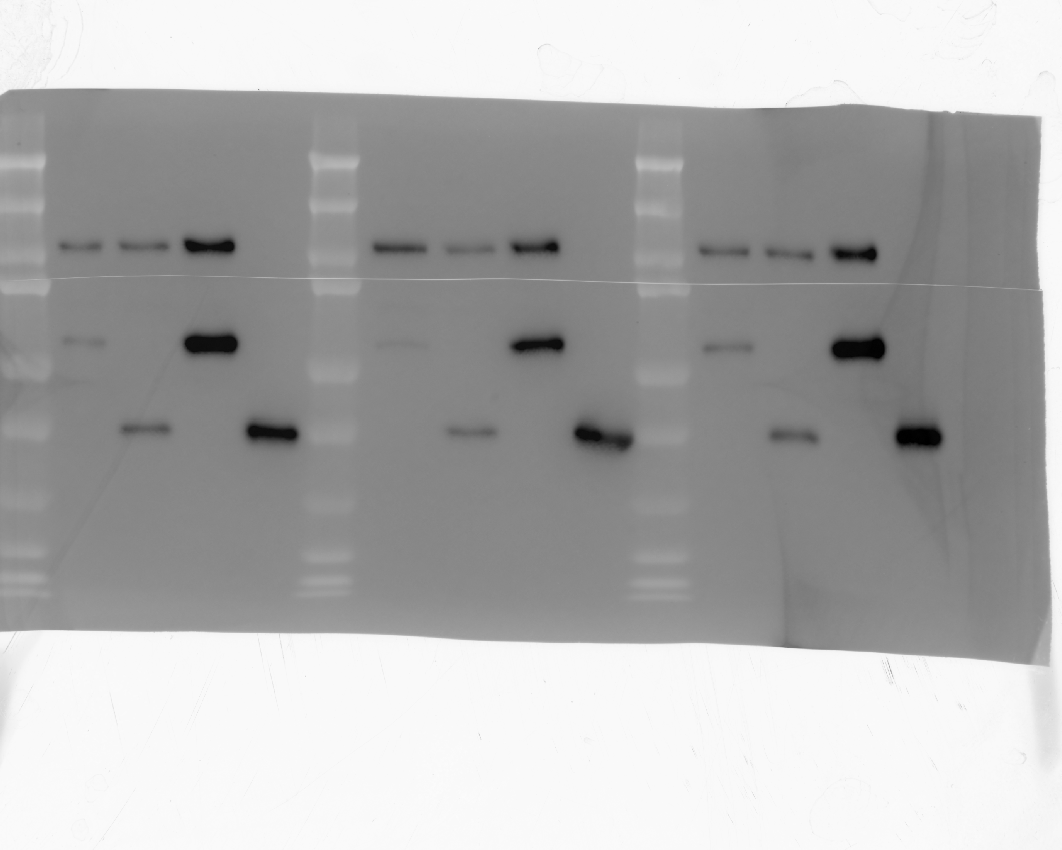

Supplement: Figure 7—source data 3. [file elife-107039-fig7-data3.zip › Figure 7-source data 3/7B.tif]
