# Supplementary material for: CCDC32 stabilizes clathrin-coated pits and drives their invagination
Source: eLife. 2026 Jan 5;14:RP107039. doi: 10.7554/eLife.107039 (PMC12768407; doi:10.7554/eLife.107039)
Supplement: Figure 2—source data 2. [file elife-107039-fig2-data2.zip › Figure 2-Source Data 2/Figure 2-Source Data 2.pdf]

2A

Vinculin、CCDC32

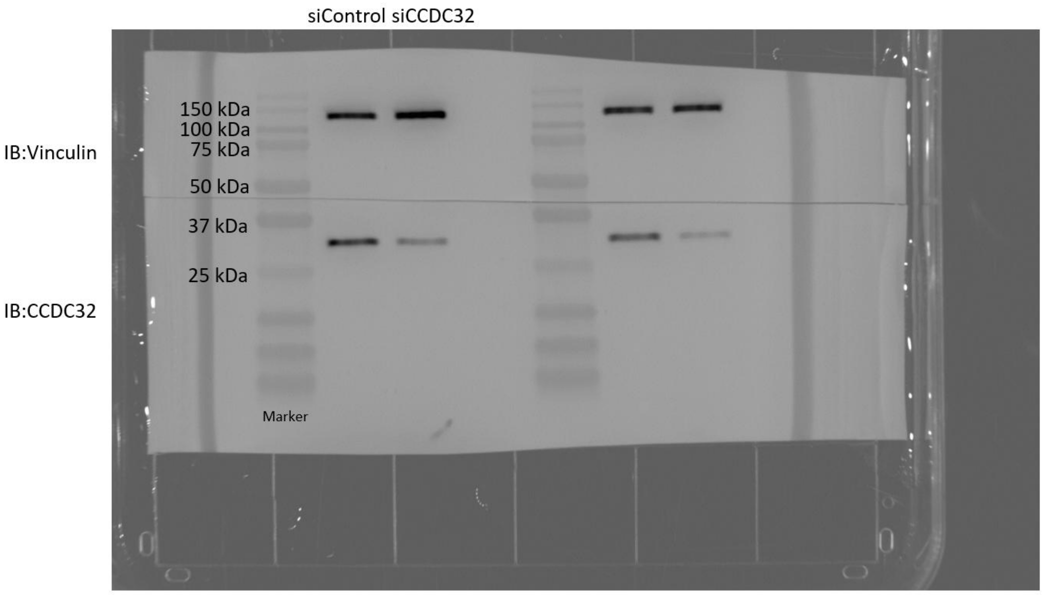

Figure 2

(A) Immunoblotting (IB) shows efficient CCDC32 knockdown in ARPE-HPV cells by siRNA treatment.
