# Supplementary material for: CCDC32 stabilizes clathrin-coated pits and drives their invagination
Source: eLife. 2026 Jan 5;14:RP107039. doi: 10.7554/eLife.107039 (PMC12768407; doi:10.7554/eLife.107039)
Supplement: Figure 2—figure supplement 1—source data 2. [file elife-107039-fig2-figsupp1-data2.zip › Figure 2-figure supplement 1-source data 2/Figure 2-figure supplement 1-source data 2.pdf]

supplement 1A

Vinculin

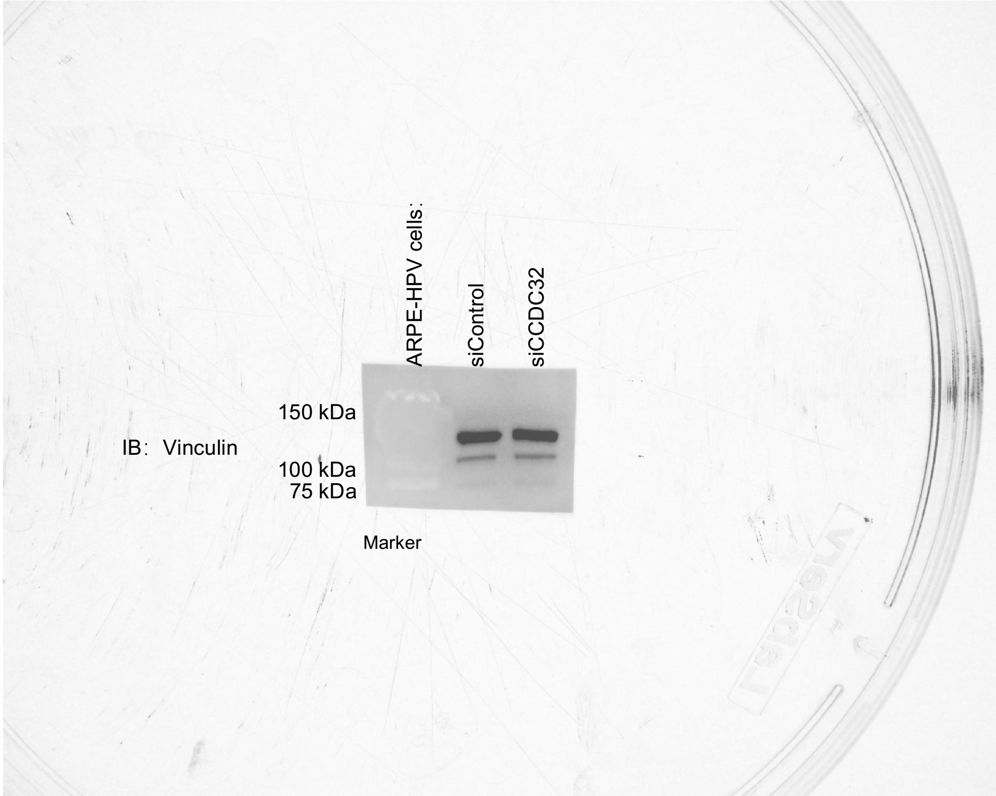

AP2-α

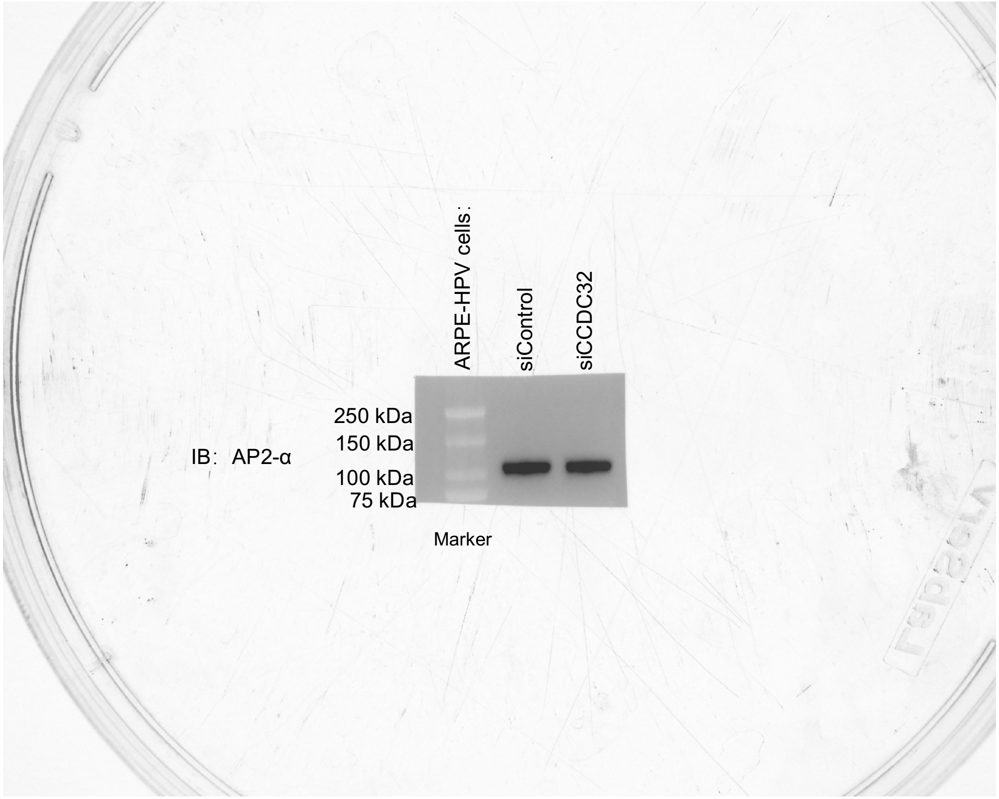

AP2- β

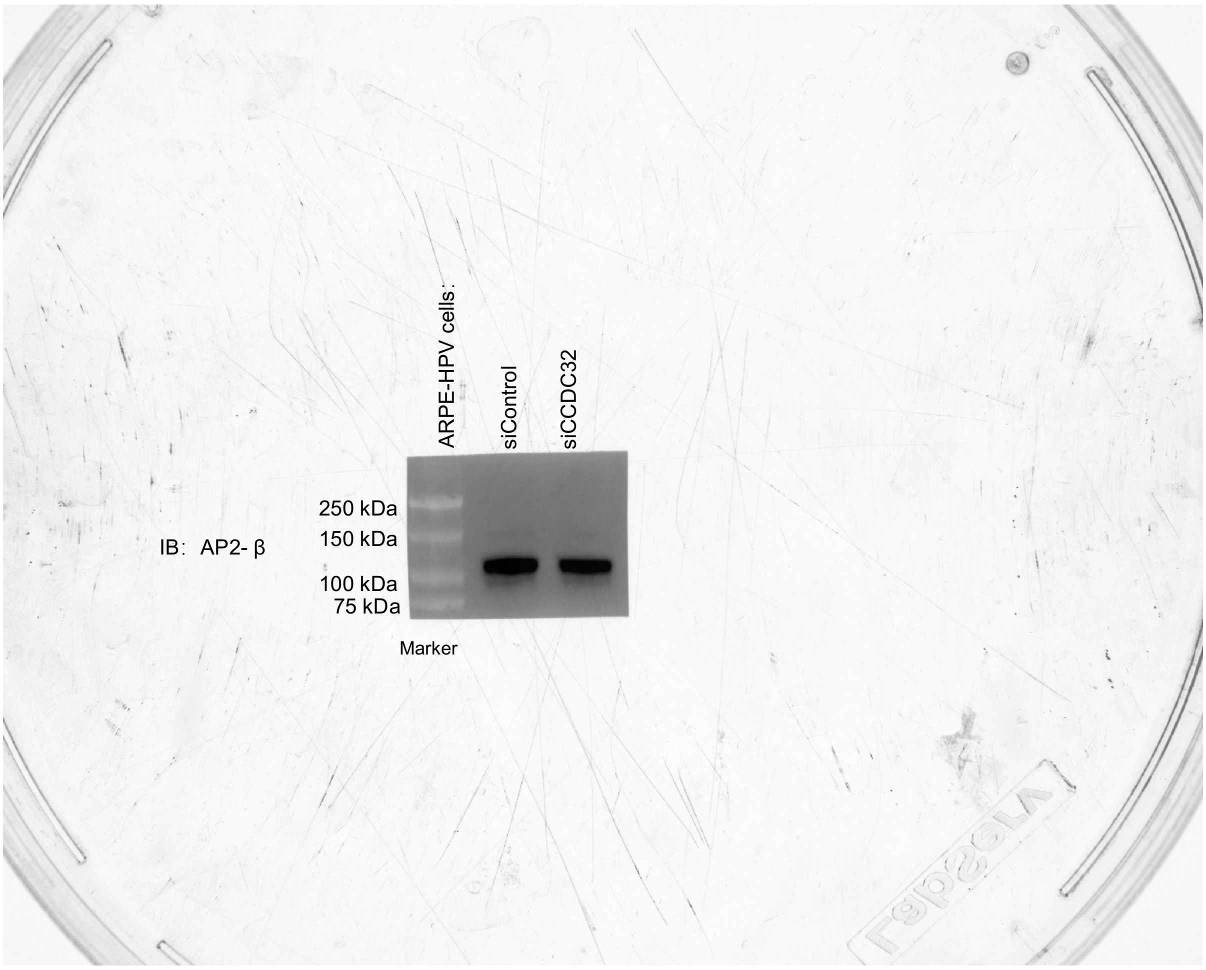
ARPE-HPV cells:
siControl
siCCDC32
250 kDa
150 kDa
100 kDa
75 kDa
IB: AP2- β
Marker

AP2- μ

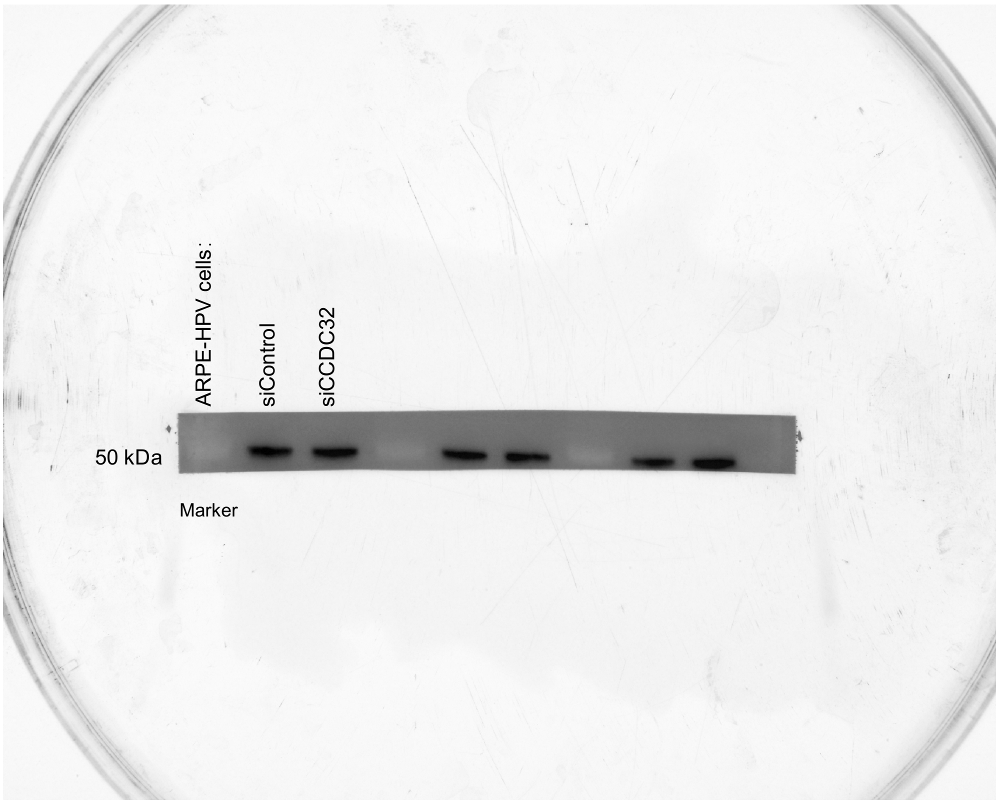
ARPE-HPV cells:
siControl
siCCDC32
50 kDa
Marker

Ap2-σ

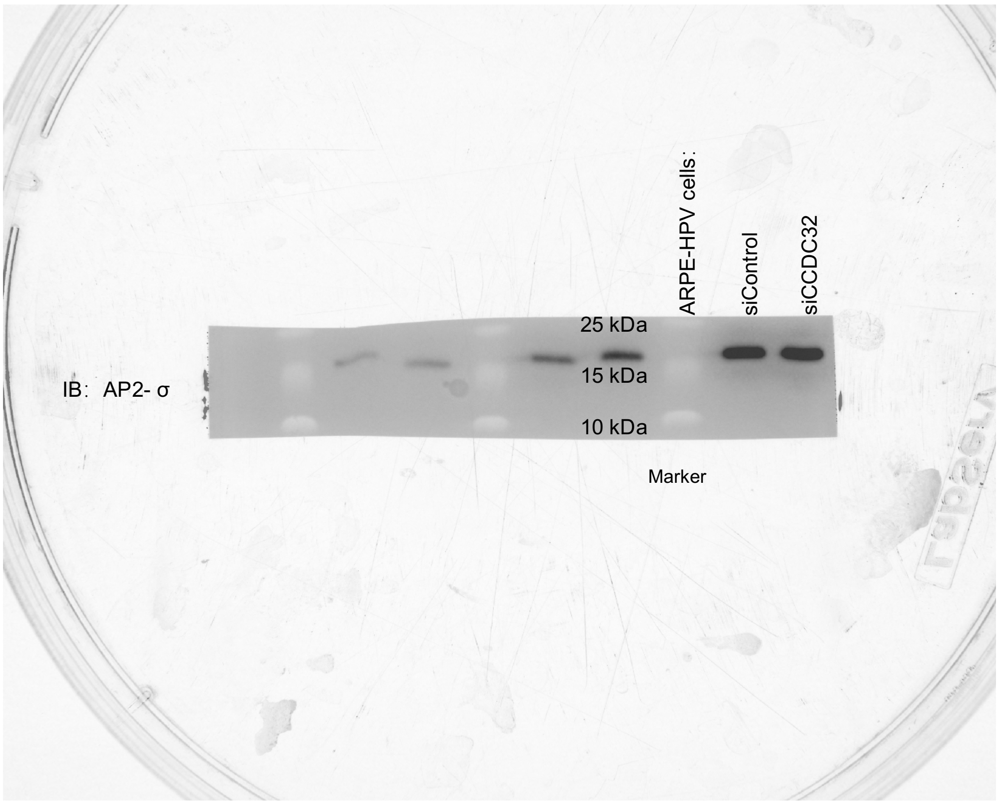

supplement 1

(A) Representative immunoblotting result of AP2 subunits from n=3 biological repeats.Error bars indicate standard deviations. Statistical analysis is student's t-test: ns, not significant.
