# Supplementary material for: CCDC32 stabilizes clathrin-coated pits and drives their invagination
Source: eLife. 2026 Jan 5;14:RP107039. doi: 10.7554/eLife.107039 (PMC12768407; doi:10.7554/eLife.107039)
Supplement: Figure 5—source data 3. [file elife-107039-fig5-data3.zip › Figure 5-source data 3/Figure 5-source data 3.pdf]

5E

AP2-α、GFP

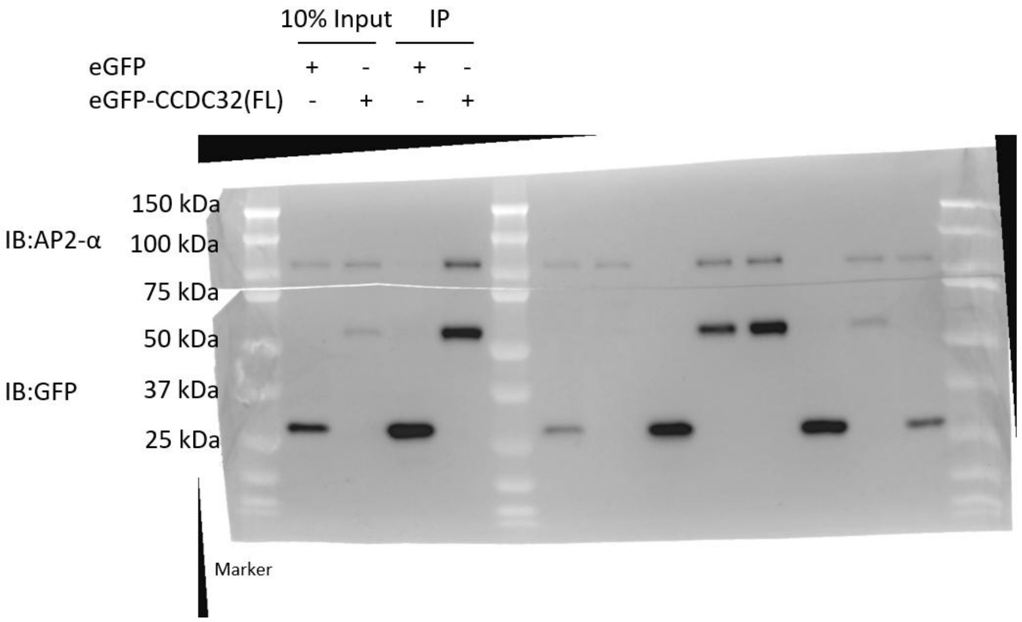
10% Input
IP
eGFP
+ - + -
eGFP-CCDC32(FL)
- + - +
150 kDa
100 kDa
75 kDa
50 kDa
37 kDa
25 kDa
IB:AP2-α
IB:GFP
Marker

5G

Coomassie stained

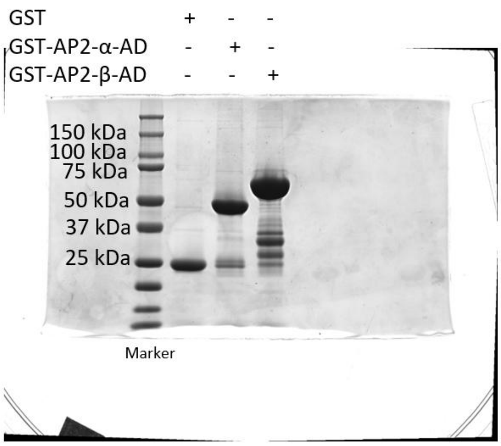

5H

GFP

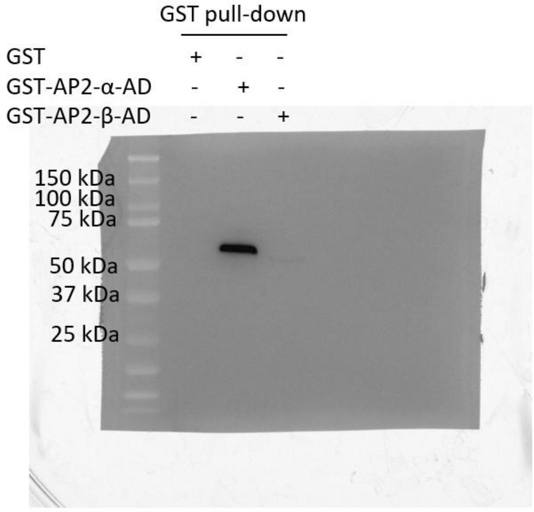

Figure 5

(E) Representative immunoblotting result of n=3 IP samples.

(G) Quantification of immunoblots of the relative enrichment of CCDC32.

(H) Coomassie blue stained SDS-page gel of purified GST, GST-AP2-α-AD, and GST-AP2-β-AD.
