# Supplementary material for: CCDC32 stabilizes clathrin-coated pits and drives their invagination
Source: eLife. 2026 Jan 5;14:RP107039. doi: 10.7554/eLife.107039 (PMC12768407; doi:10.7554/eLife.107039)
Supplement: Figure 5—figure supplement 1—source data 1. [file elife-107039-fig5-figsupp1-data1.zip › Figure 5-figure supplement 1-source data 1/Figure 5-figure supplement 1-source data 1.pdf]

supplement 1

AP2-α

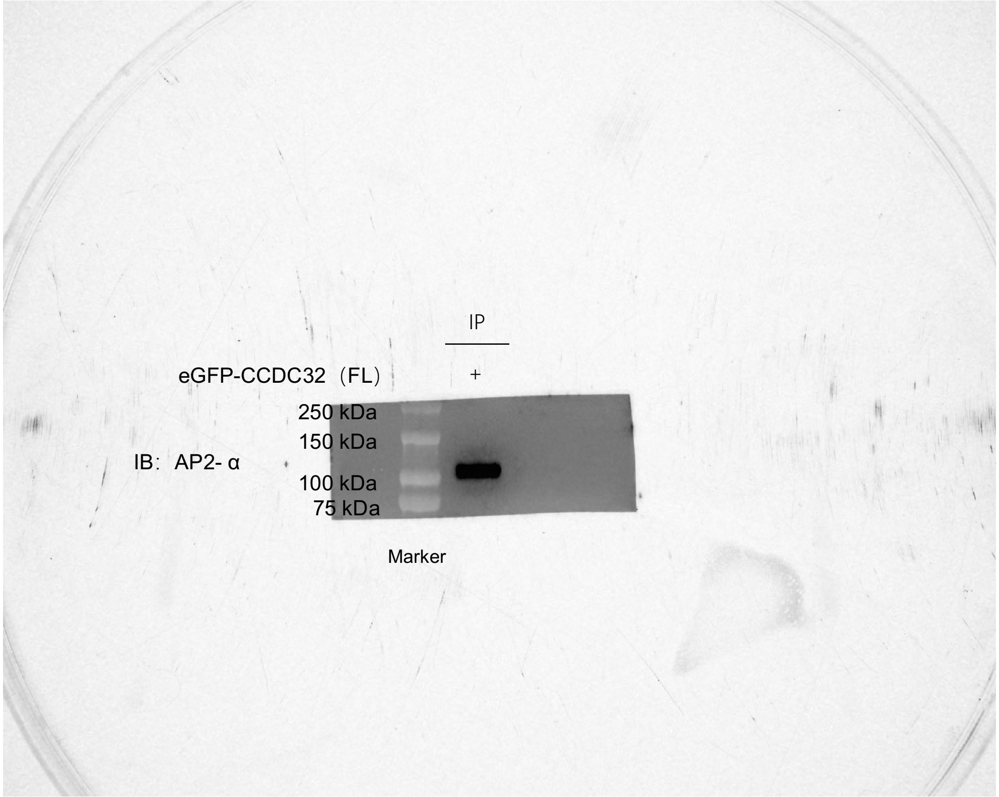

AP2- β

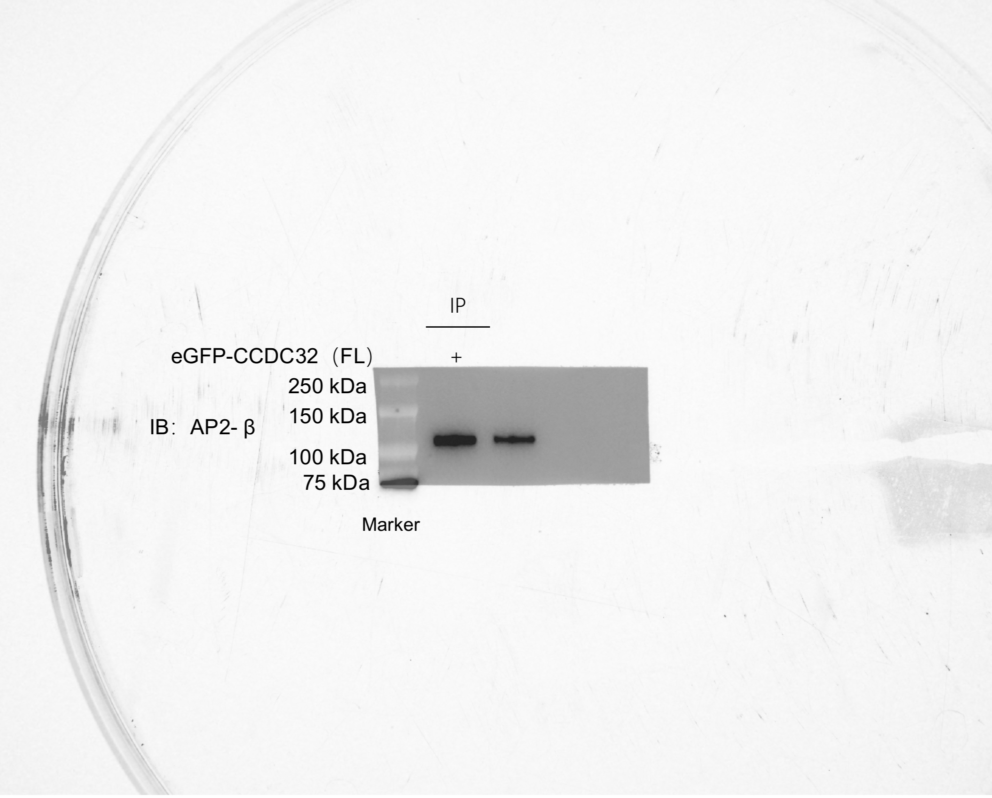

AP2- μ

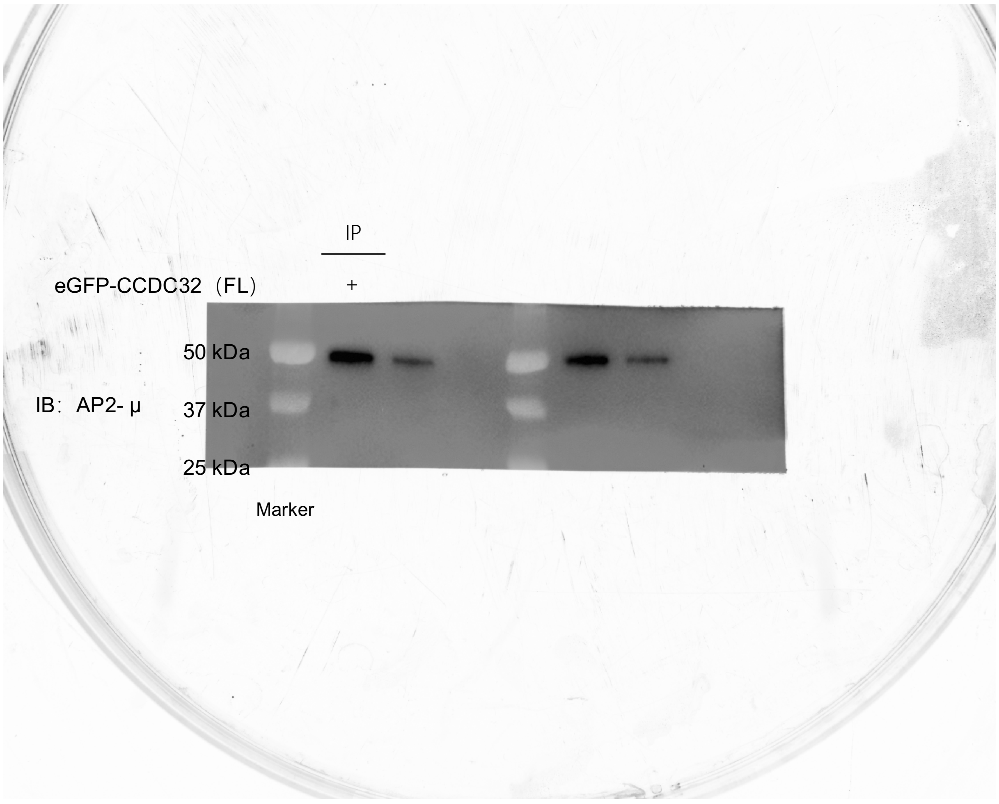
IP
eGFP-CCDC32（FL）
+
50 kDa
IB：AP2- μ
37 kDa
25 kDa
Marker

Ap2-σ

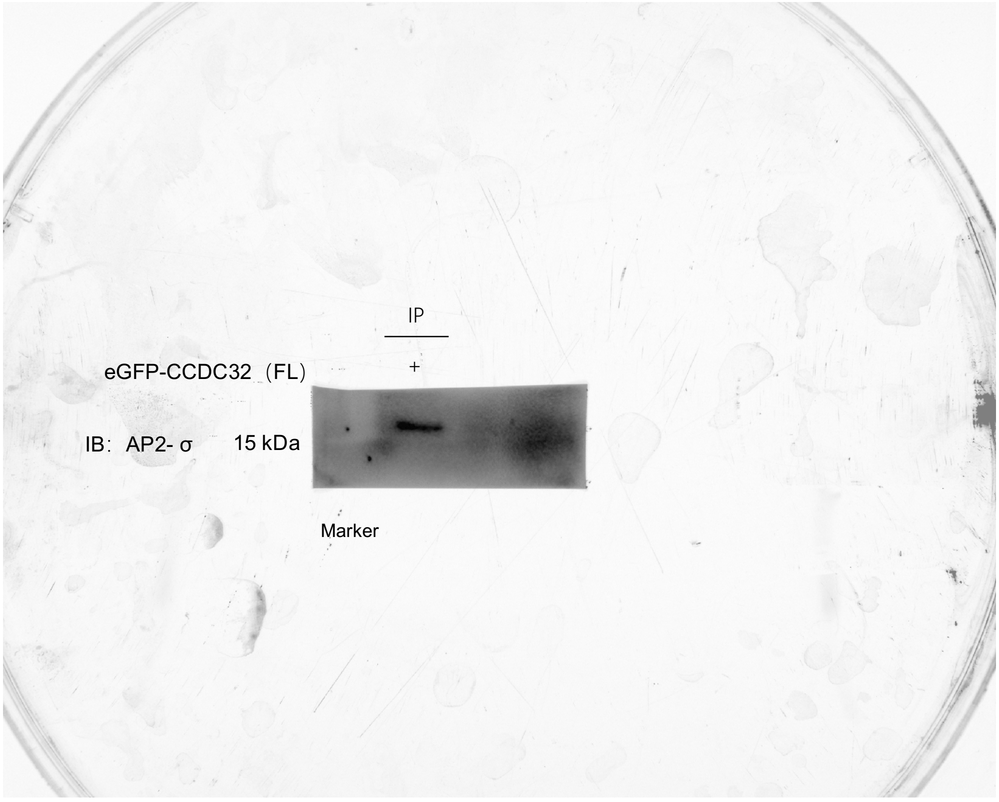
IP
eGFP-CCDC32（FL）
+
IB：AP2- σ
15 kDa
Marker

Figure supplement 1

All the four subunits of intact AP2 efficiently co-IP with eGFPCCDC32(FL).
