# Supplementary material for: CCDC32 stabilizes clathrin-coated pits and drives their invagination
Source: eLife. 2026 Jan 5;14:RP107039. doi: 10.7554/eLife.107039 (PMC12768407; doi:10.7554/eLife.107039)
Supplement: Figure 6—source data 2. [file elife-107039-fig6-data2.zip › Figure 6-source data 2/Figure 6-source data 2.pdf]

6B-left

AP2-α、GFP

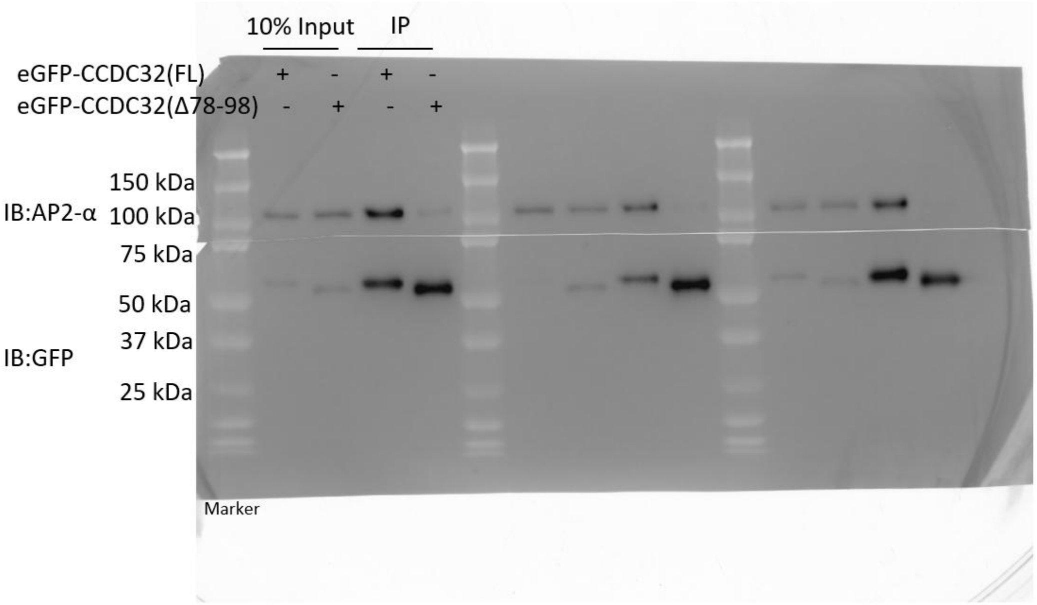

6B_right

AP2-β

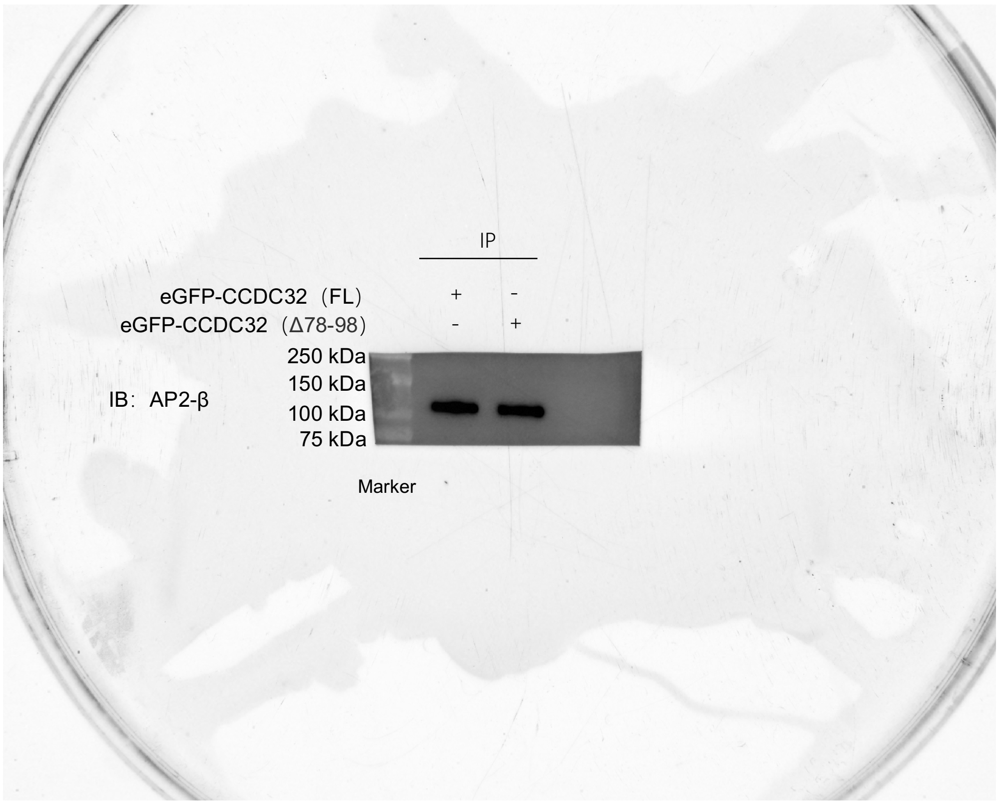

AP2- μ

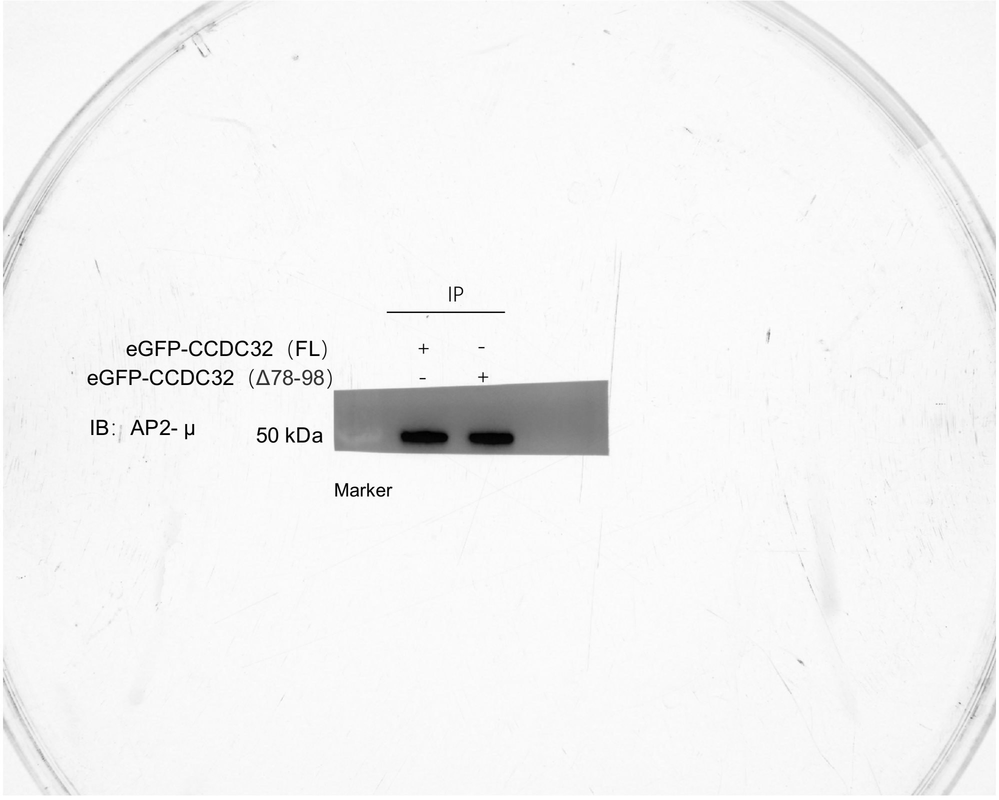

Ap2-σ

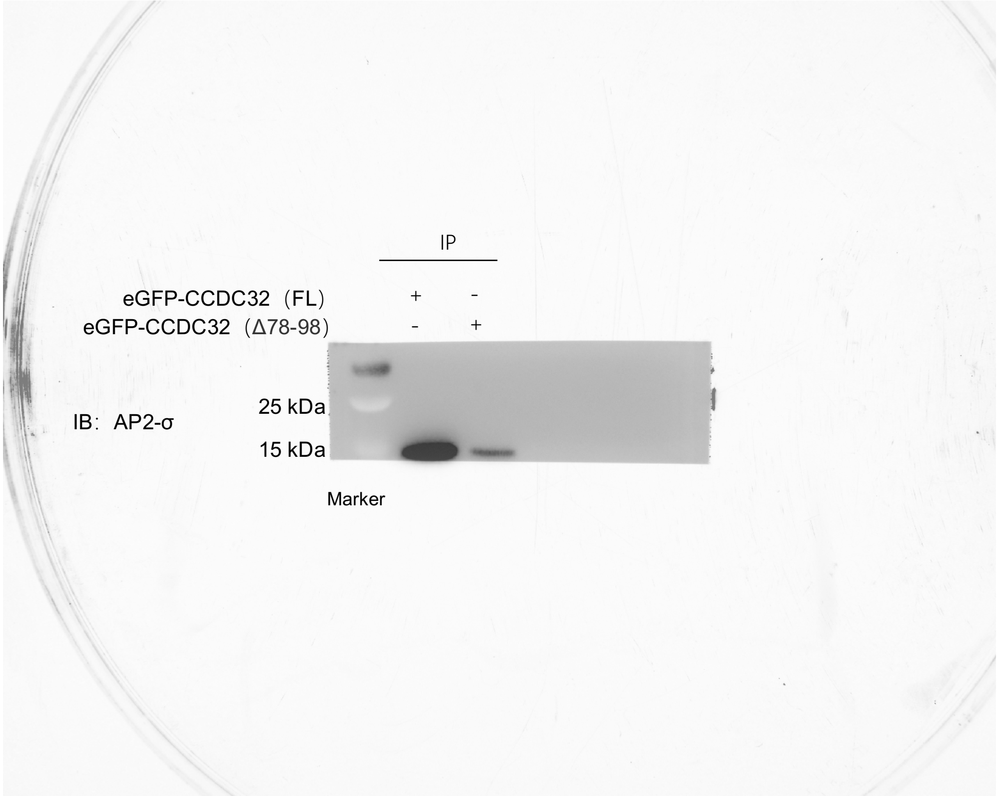

Figure 6

(B) Representative Immunoblotting result of n=3 IP samples.
